# Three-dimensional characterization of fibrous microstructure in meat analogs using micro-computed X-ray tomography/μCT-based granulometry

**DOI:** 10.3389/fnut.2026.1843070

**Published:** 2026-07-22

**Authors:** Xinyu Pan, Tiaan Friedrich, Michelle Müller, Magdalena Bock, Jan Engmann, Guilherme De Oliveira Reis, Mark Ambühl, Christoph Hartmann, Heiko Briesen

**Affiliations:** 1 Process Systems Engineering, Technische Universität München, Freising, Germany; 2 Société des Produits Nestlé S.A., Lausanne, Switzerland; 3 Technische Universität Berlin, Berlin, Germany; 4 Ecole Polytechnique Fédérale de Lausanne, Lausanne, Switzerland

**Keywords:** 3D microstructure, anisotropy, characteristic size, fiber orientation, granulometry, meat analog, micro-computed X-ray tomography

## Abstract

The fibrous microstructure of plant-based meat analogs governs their textural properties, yet quantitative 3D characterization remains limited. In this study, a granulometry-based approach was extended to 3D micro-computed X-ray tomography (μCT) datasets to characterize the fiber architecture of high-moisture extruded soy protein concentrate meat analogs. Five prototype samples, produced at extrusion temperatures from 90°C to 170 °C, and three commercial products were analyzed for characteristic fiber width and length, orientation distribution, anisotropy, and shape metrics. The characteristic fiber width increased progressively with extrusion temperature, whereas anisotropy first increased and then decreased, indicating an optimal temperature window for fiber alignment. The commercial samples exhibited coarser structures with lower anisotropy, attributed to more complex ingredient composition. The proposed approach provides a comprehensive quantitative framework for evaluating fibrous microstructures in extruded plant-based meat analogs.

## 1 Introduction

The global shift toward plant-based diets is increasingly recognized for substantial benefits to human health, environmental sustainability, resource efficiency, and animal welfare (1–3). Driven by growing consumer awareness of environmental, ethical, and health-related concerns, the global market for plant-based meat alternatives has experienced sustained growth since 2018, with a compound annual growth rate (CAGR) of 13.55% between 2018 and 2025 (4, 5). In 2025, the market revenue for meat substitute products is estimated at approximately USD 11.34 billion. According to market projections, this value is expected to increase further to USD 17.05 billion by 2030, indicating continued expansion of the sector (5).

The texture of meat analogs is strongly governed by directional fibrous structures, which determine both their mechanical anisotropy and visual appearance (6–9). A pronounced, well-oriented fibrous architecture promotes a meat-like bite because aligned fibers and their interconnections produce distinct mechanical responses when the product is loaded parallel versus perpendicular to the fiber direction (9–11).

To quantify this structural effect, mechanical anisotropy is widely used as a descriptor of fiber formation in meat analogs. It is commonly defined as the ratio of forces required to cut or stretch the product parallel and perpendicular to the visible fiber orientation, or, for extrudates, relative to the extrusion flow direction at the die exit (7, 10) demonstrated strong correlations between macrostructural fiber scores and mechanical anisotropy-related metrics in meat analogs.

Imaging technologies play a central role in investigating the structural characteristics of meat analogs and include visual, spectroscopic, and microscopic techniques (8). Ma et al. (12) developed Fiberlyzer, an automated image-analysis tool that enables quantitative assessment of the visual fibrousness of meat analogs. The method quantifies structural descriptors, including anisotropy (defined as the ratio of fiber length to fiber width), fiber area, and the number of fiber branches. In addition, microscopic techniques such as scanning electron microscopy (SEM) and confocal laser scanning microscopy (CLSM) are frequently employed to analyze the microstructure of meat analogs (9, 11, 13, 14).

However, these techniques are inherently limited in their ability to capture the three-dimensional fiber architecture. In contrast, X-ray-based imaging methods provide a non-invasive means of visualizing internal three-dimensional structures without the need for staining or microtome sectioning. In meat analog research, X-ray microscopy (XRM) has been applied to quantify pore-network characteristics, including porosity, connectivity, and surface area, in textured protein scaffolds (15), and X-ray tomography (XRT) has been used to analyze layered microstructures in frozen extrudates (16). As a micrometer-scale resolution is typically aimed for, the method is commonly referred to as micro-computed X-ray tomography, or in short μCT.

Despite these advances, only a limited number of studies have quantitatively analyzed fiber orientation, fiber length, and layer thickness in extruded meat analogs. Auer et al. (6) used structure tensor analysis to investigate fiber orientation and reported that two-dimensional light microscopy suggested a more pronounced orientation, whereas three-dimensional micro-CT analysis revealed a largely isotropic fiber distribution.

Comparable challenges in fiber characterization are well known in composite materials research, where fiber orientation governs the mechanical properties of composite materials (17–20). In this context, Gager et al. (17) introduced an original method based on gray-level granulometry to analyze the fiber orientation distribution in composite reinforcements.

In the present study, the granulometry-based approach is extended to three-dimensional μCT datasets to enable a volumetric characterization of fiber morphology in extruded meat analogs. Characteristic fiber width, length, orientation distribution, anisotropy, and shape metrics are analyzed for both prototype samples produced at varying texturization maximum barrel temperatures and commercial products. The method’s accuracy is validated using synthetically generated structures with known dimensions and orientations. The aim is to provide a versatile 3D image analysis toolbox for quantifying morphological differences in fibrous extrudate structures, which may serve as a foundation for future investigations linking microstructural features to processing parameters and mechanical properties.

## 2 Materials and methods

### 2.1 Samples

Soy protein was selected because it is the most widely used protein source in meat analogs. Three commercial soy-based samples and five soy-based prototype samples were analyzed (Table 1). Three commercial plant-based meat analogs were purchased from REWE supermarkets in Freising, Germany. For the commercial samples compositional information was identified from the declaration. However, detailed processing conditions were unknown. By contrast, the prototype samples consisted exclusively of soy protein concentrate (SPC) and water, which gave them a substantially higher protein content than the commercial products. The prototype samples were produced by high-moisture extrusion and provided by Nestlé. Without disclosing detailed extruder geometry, the main differences of the samples were the temperature profile along the extruder as denoted in Table 1. As it was not the purpose of this study to correlate extrudate structure to processing conditions or composition in detail neither of them is relevant. The samples have only been chosen to probe a variety of different structures to support the generalization of the proposed granulometric characterization.

**Table 1:** Sample ingredient lists as provided by the manufacturers.

Sample type	Sample number	Ingredients					
Commercial samples	Commercial sample 1: Butcher Schnitzel	Drinking water, 22% soy base (water, soy protein, wheat gluten, starch), 21% breadcrumbs (wheat flour, wheat fiber, yeast, salt), sunflower oil, fibers (oat, psyllium), soy protein, wheat flour, thickener: methylcellulose, shea butter, natural flavor, starch, modified wheat starch, spirit vinegar, coconut oil, salt, acidifier (lactic acid), pepper, ferric diphosphate, vitamin B12					
	Commercial sample 2: Garden Gourmet	Drinking water, 11.8% soy protein, flour (wheat, rice), 5.3% onions, vegetable oils (sunflower, rapeseed), 3.7% wheat protein, yeast extract, starch, spices (onion powder, garlic, pepper, turmeric), apple purée, spirit vinegar, lemon fiber, fried onions, salt, flavors (with celery), pea fiber, pH regulator (potassium hydroxide)					
	Commercial sample 3: Mühlen Schnitzel	Drinking water, wheat flour, 11% soy protein, rapeseed oil, wheat gluten, salt, thickener: methylcellulose, corn flour, natural flavor, wheat starch, spirit vinegar, spices, sugar, psyllium husk, yeast					
Sample type	Sample number	Ingredients	Barrel temperature (°C)				Cooling die temperature (°C)
			Zone 1	Zone 2	Zone 3	Zone 4–12	
Prototype samples	1	SPC (67% protein), 55% moisture (w/w)	40	80	90	90	60
	2					120	
	3					130	
	4					150	
	5					170	

To capture their three-dimensional structure, all samples were cut into cubic specimens with an edgelength of approximately 1 cm. The samples were not cooked but used straight from the package. The specimens were stored at −18 °C and subsequently freeze-dried using a laboratory freeze-dryer (Alpha 2–4 LSCplus, Martin Christ, Germany). Freeze-drying was used to remove water and improve X-ray contrast between the plant-protein matrix and air-filled pores, thereby facilitating segmentation and microstructural analysis. No appreciable volumetric changes were observed after freeze-drying, as indicated by the absence of visible macroscopic deformation, shrinkage, or collapse. Although freeze-drying may slightly alter the micro-porosity of the samples due to ice crystal formation, it is known to preserve the macroscopic geometry (21–25). Since all samples were subjected to identical freezing and drying conditions, any such effects are systematic and do not compromise relative comparisons between samples.

After freeze-drying, the prototype and commercial samples were prepared differently for μCT scanning. The commercial samples were glued onto a sample holder and scanned as whole specimens. In contrast, the prototype samples, which became brittle after freeze-drying due to their layered texture, could be easily chipped into smaller pieces and placed in a sample holder tube with an inner diameter of 5.75 mm. The samples were then scanned using a micro-computed tomography (μCT) scanner (CT-1600HR, MatriX Technologies GmbH, Feldkirchen, Germany). Commercial samples were scanned at a resolution of 8 μm, an accelerating voltage of 60 kV, a tube current of 60 mA, and an exposure time of 1,200 ms. In contrast, the Prototype samples were scanned at a higher resolution of 4 μm, a voltage of 80 kV, a current of 50 mA, and an exposure time of 1,200 ms. Because the prototype samples had higher protein content and formed densely packed protein networks, a higher voxel resolution was required to reliably resolve fine structural features. The number of projections was 2000 for each scan, and the data were reconstructed using MatriX CT Reconstruction v5.1.6.0 (Nordson Matrix Technologies GmbH, Feldkirchen, Germany) to produce a 2000 × 2000 × 1,225 voxel dataset.

### 2.2 Image analysis methodology

To localize the sample and reduce the surrounding background, the image volumes were binarized using Otsu’s method (26), an automatic thresholding approach that determines the optimal intensity threshold by maximizing the inter-class variance between foreground and background. The largest connected component was then extracted to mask the background. The resulting masked images were converted to 8-bit grayscale and histogram-equalized to enhance contrast. Pixel intensities were then standardized to a [0, 1] range using quantile clipping and normalization. Next, to remove excess background volume, the 3D volume was rotated to align one of the physical edges of the cubic sample horizontally and then cropped to its bounding box.

A voxel-wise solidity map was then computed from the binary volume using a 3D box filter with a kernel size of 101 × 101 × 101 voxels (imboxfilt3, MATLAB), where the solidity at each voxel represents the local solid volume fraction within its neighborhood. The volume was subsequently partitioned into non-overlapping sub-volumes of 200 × 200 × 200 voxels, and the mean solidity was calculated for each sub-volume. The edge length of 200 was chosen to be approximately 10 times the width of the usual fiber. This ensures that there are enough fibers available locally to capture spatial trends, while keeping memory requirements to a minimum. To ensure that the selected regions contained sufficient internal structure, the 50 sub-volumes with the highest mean solidity values were retained. This sampling procedure ensures that the impact of the outermost regions, which may be affected by artifacts resulting from the preparation process, is excluded from the final analysis. For granulometric analysis, 15 sub-volumes were randomly selected from this pool, and this randomized selection was repeated independently five times to evaluate the structural homogeneity of each sample.

To compute the local fiber orientation at each voxel, we adopted the method proposed by Gager et al. (17), which is based on gray-level granulometry using directional morphological operations. In gray level granulometry, the orientation of fibrous structures can be determined by applying morphological operations with structuring elements of varying length and orientation (17). The analysis is based on the application of morphological opening operators to two- or three-dimensional grayscale images, using structuring elements of increasing length ( $SE_{1}$ , $SE_{2}$ ,𝑒𝑡𝑐). The choice of operator depends on the intensity characteristics of the feature of interest; for instance, bright fibers are evaluated using opening operations. Following the iterative application of the morphological operator with increasing structuring element lengths, an image volume curve $V_{i}$ is generated for each step, representing the sum of voxel intensities within the processed image. By normalizing and differentiating this volume curve, the granulometric value *G*_*i*_ (Equation 1) is calculated, quantifying the relative intensity change between consecutive structuring element lengths (17, 27).


$$G_{i}=\frac{V_{i}-V_{i+1}}{V_{0}-V_{n}}$$ (1)

Where $V_{i}$ denotes the summed voxel intensity after processing with structuring element length $SE_{i}$ , $V_{i+1}$ the summed voxel intensity after processing with $SE_{i+1}$ , $V_{0}$ the initial summed intensity of the original image, $V_{n}$ the final summed intensity after $SE_{\max}$ , and $G_{i}$ the relative change in structure content between lengths $SE_{i}$ and $SE_{i+1}$ .

Morphological operations can be conceptually compared to sieving processes, as increasing the length of the structuring element leads to the progressive removal of image features, depending on whether they are darker or lighter than the background. The resulting granulometric curve thus serves as an indicator of the particle size distribution in the image domain (27).

Beyond size characterization, granulometry can also be extended to assess structural orientation. This involves applying directional structuring elements, such as elongated rods or elliptical shapes, with systematically varied orientations and sizes. For each orientation Ω(θ,α) , where θ denotes the azimuth angle and α the elevation angle, and for each length 𝑥 of $SE_{i}(\Omega ,x)$ , a granulometric curve $G_{i}(\Omega ,x)$ is calculated, from which the geometric mean size $m_{G}(\Omega ,x)$ (Equation 2) can be derived to represent the predominant structure size (17):


$$m_{G}(\Omega ,x)=\exp(\sum_{i}^{\max}\log(SE_{i}(\Omega ,x)).G_{i}(\Omega ,x))$$ (2)

Anisotropic structuring elements $\left(SE_{i}(\Omega ,x)\right)$ were designed as a series of line-shaped elements with varying orientations and lengths. To generate the orientations, a geodesic sphere discretization was employed, starting from a regular icosahedron consisting of 12 vertices and 20 triangular faces. The triangular mesh was iteratively refined by subdividing each face and projecting the newly created vertices onto the surface of the unit sphere, thereby ensuring an approximately uniform angular distribution of orientations.

Since fiber orientation is direction-independent with respect to axial sign (i.e., ± directions are equivalent), only the upper hemisphere was retained. The number of orientations generated on this half-sphere depended on the refinement level: two refinement iterations yielded 89 distinct orientations, whereas three iterations produced 337. The final orientations were defined in spherical coordinates, combining azimuth angles within the xy-plane and elevation angles relative to the z-axis. For each orientation, morphological opening operations were applied to the image volume using increasing lengths, ranging from 16 μm to 320 μm (in steps of 16 μm).

The accuracy of the granulometric analysis depends on the number of employed structuring elements. Increasing the number of orientation refinements and the number of length steps improves the angular and length resolution of the analysis, thereby enabling a more accurate characterization of fiber length and orientation. However, this improvement is accompanied by increased computational costs; therefore, an appropriate trade-off between accuracy and efficiency must be selected.

### 2.3 Fiber structural characterization

**Figure 1:**
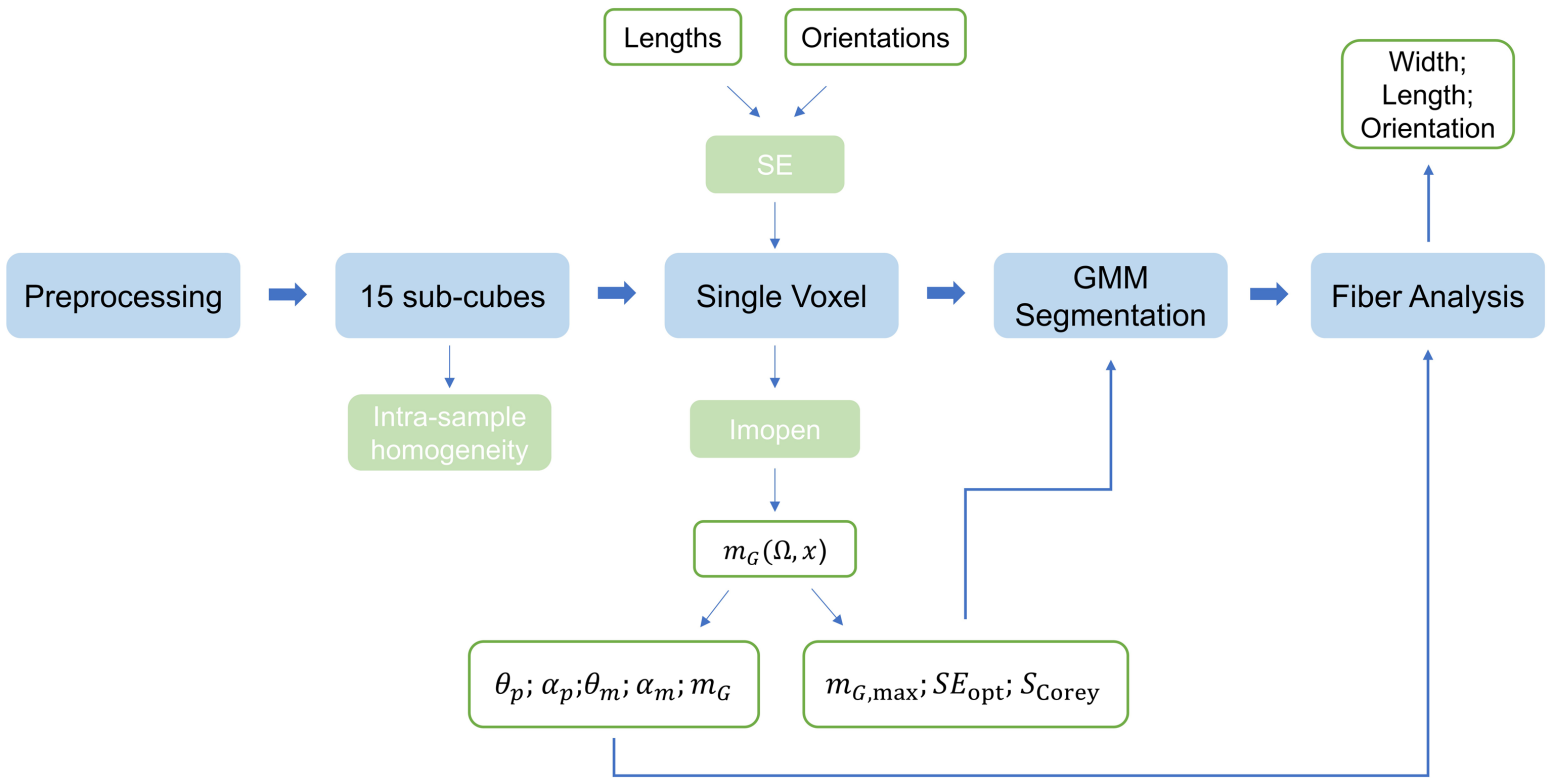
Workflow of the fiber structure analysis following five sequential steps: (1) preprocessing of the image data; (2) selection of 15 sub-cubes from the 50 candidates with the highest solidity; (3) morphological opening (imopen) using line-shaped structuring elements with different lengths and orientations; (4) GMM-based segmentation using three voxel-wise descriptors: the maximum granulometric mean size (*m*_*G*_,_max_), the Corey shape factor (*S*_Corey_), and the opened-image intensity obtained with the optimal structuring element (*SE*_opt_); and (5) fiber analysis yielding length and width from the principal axis (*p*) and minor axis (*m*) of the directional granulometric mean-size distribution, with the final orientation parameters derived from the corresponding axis angles (*θ*_*p*_), (*α*_*p*_), (*θ*_*m*_), and (*α*_*m*_).

The anisotropic granulometry produced, for each voxel, a set of directional and scale-dependent responses obtained from line-shaped structuring elements with varying orientations and lengths. From these responses, granulometry-derived descriptors were assembled, including (i) the maximum granulometric mean size *m*_*G,*__max_, (ii) the morphologically opened-image intensity *V*_*i*_, obtained using the optimal structuring element *SE*_opt_, where *SE*_opt_ denotes the structuring element whose associated granulometric mean size corresponds most closely to *m*_*G,*__max_ and (iii) a shape-based anisotropy factor *S*_Corey_ derived from the response distribution. The workflow is visualized in Figure 1.

These voxel-wise features were first used for probabilistic material segmentation via a two-stage Gaussian Mixture Model (GMM). In the first step, a two-component GMM separated foreground from background voxels. The background component was identified based on the mixture proportions, and all remaining voxels were retained as foreground.

After this GMM-based segmentation, subsequent microstructural characterization was restricted to voxels classified as fibers. Within the fiber mask, the dominant local fiber orientation was obtained from the granulometric response distribution and expressed as azimuth and elevation angles. In addition, characteristic length and width scales were extracted from an equivalent ellipsoidal representation of the directional response distribution via eigenvalue decomposition, yielding a principal characteristic length (dominant structural extension) and a minor characteristic length (transverse/minimal detectable dimension). This ellipsoidal representation therefore enables the local distribution of granulometric lengths to be quantified into three lengths and their three accompanying orientations in a numerically efficient manner. Based on the characteristic length, also the local fiber shape can be evaluated. A common measure of overall shape is the Corey shape factor, which is the ratio of the shortest characteristic length, divided by the square root of the product of the remaining two lengths (28). This shape factor is close to 1 for spherical local shapes, decreasing with elongated shapes and lowest for plate-like shapes (28).

Additionally, fiber anisotropy was quantified from the eigenvalues of the normalized inertia tensor computed for fiber voxels. The formulation of the anisotropy index in Equation 3 is similar to the work of Schröder-Turk (29), with an additional scaling, such that higher values denote higher anisotropies. Anisotropy was further characterized using the Fractional Anisotropy (FA) metric, originally introduced for diffusion tensor imaging (30). FA (Equations 4,5) has been widely used to assess the alignment of white-matter fiber tracts (31) and can be analogously applied to quantify the degree of alignment in fiber-based microstructures, where $\lambda_{1}\geq \lambda_{2}\geq \lambda_{3}$ are the ordered eigenvalues of the second-moment tensor.


$$\text{Anisotropy}=1-\frac{\lambda_{3}}{\lambda_{1}}$$ (3)

$$\mathrm{FA}=\sqrt{\frac{3}{2}\frac{{\left(\lambda_{1}-\bar{\lambda }\right)}^{2}+{\left(\lambda_{2}-\bar{\lambda }\right)}^{2}+{\left(\lambda_{3}-\bar{\lambda }\right)}^{2}}{\lambda_{1}^{2}+\lambda_{2}^{2}+\lambda_{3}^{2}}}$$ (4)

$$\bar{\lambda }=\frac{1}{3}(\lambda_{1}+\lambda_{2}+\lambda_{3})$$ (5)

$$c_{p}=\frac{2(\lambda_{2}-\lambda_{3})}{\lambda_{1}+\lambda_{2}+\lambda_{3}}$$ (6)

$$c_{l}=\frac{\lambda_{1}-\lambda_{2}}{\lambda_{1}+\lambda_{2}+\lambda_{3}}$$ (7)

$$c_{s}=\frac{3\lambda_{3}}{\lambda_{1}+\lambda_{2}+\lambda_{3}}$$ (8)

We adopted the anisotropy measures (Equations 6−8) proposed by Westin et al. (32) due to their straightforward geometric interpretation. These metrics describe three different types of anisotropy: linear ( $c_{l}$ ), planar ( $c_{p})$ and spherical ( $c_{s}$ ). All metrics are normalized to the range [0, 1] and satisfy the relation $c_{p}+c_{l}+c_{s}=1$ . The ellipsoids associated with each metric illustrate the diffusion tensor shape for which the corresponding metric is dominant, while the other two metrics approach zero.

Furthermore, we applied the method introduced by Kindlmann & Weinstein (33) for Barycentric Opacity Mapping to visualize the metrics. In this representation, each vertex of the barycentric triangle corresponds to one anisotropy measure being equal to one, while the other two are zero. Along the edges of the triangle, one measure is zero while the other two sum to one. Different colors are used to visualize the barycentric space of anisotropies (see Section 3.2.4).

Using the obtained CT data, granulometry was applied to determine the characteristic length, width, and orientation of the fibers. Furthermore, fiber shape descriptors were extracted, allowing an assessment of whether the fibers form a sheet-like structure.

## 3 Results and discussion

### 3.1 Validation

To validate the orientation estimation and quantify the impact of angular discretization, synthetic fiber-like structures were generated using a 3D Bresenham line algorithm. Fibers with a fixed length of 600 voxels were created using 50 randomly sampled orientations Ω(θ,α) . The azimuth angle θ was sampled from [0, 360°], while the elevation angle α was sampled from [0, 90°].

**Figure 2:**
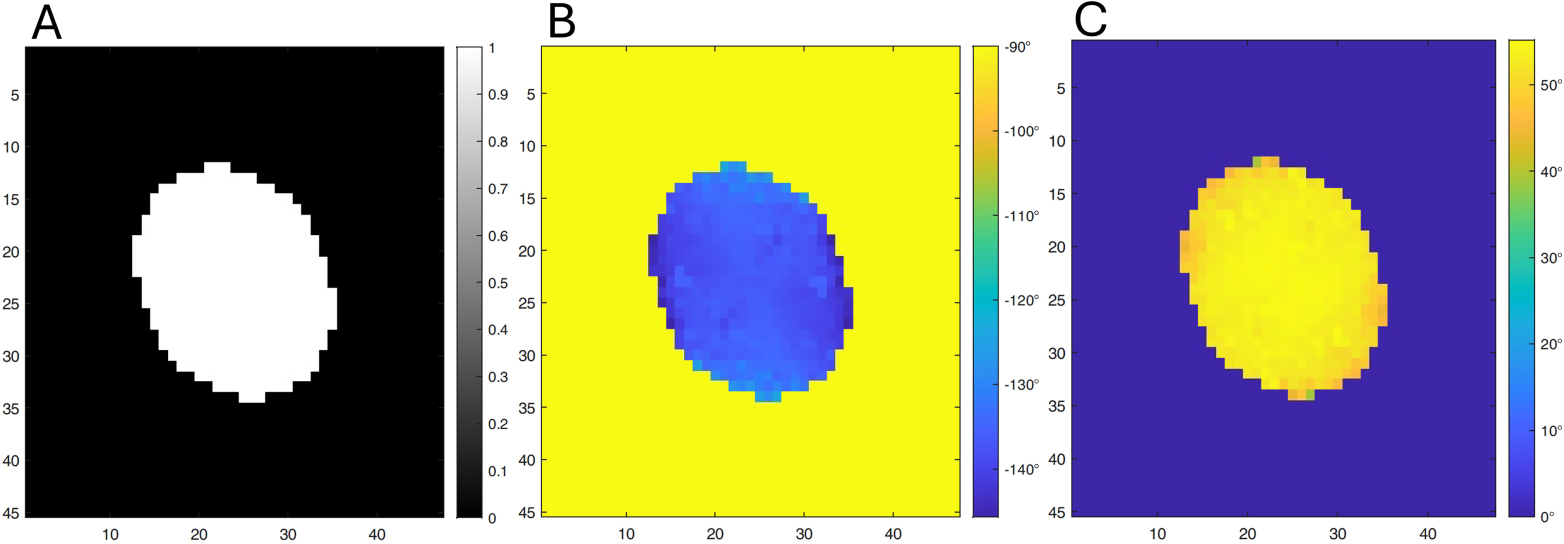
Validation of granulometric orientation estimation on a synthetic object of known orientation. A 3D synthetic object was generated with a prescribed azimuth of 33° and elevation of 47°, and its orientation was estimated by granulometry; results are shown for a representative 2D slice. **(A)** Representative 2D slice extracted from the 3D synthetic object; the color bar gives the grayscale value of the synthetic object (0–1). **(B)** Estimated azimuth orientation map for the same slice; color bar in degrees (~−140° to −90°). **(C)** Estimated elevation orientation map for the same slice; color bar in degrees (0° to ~55°).

These structures were analyzed via anisotropic granulometry across two discretization levels: a two-iteration geodesic sphere (87 orientations) and a three-iteration discretization (337 orientations). As illustrated in Figure 2, the slice-wise orientation maps for a fiber with prescribed angles ( θ =33°, α =47°) show that the estimated dominant orientations align closely with the ground truth.

**Figure 3:**
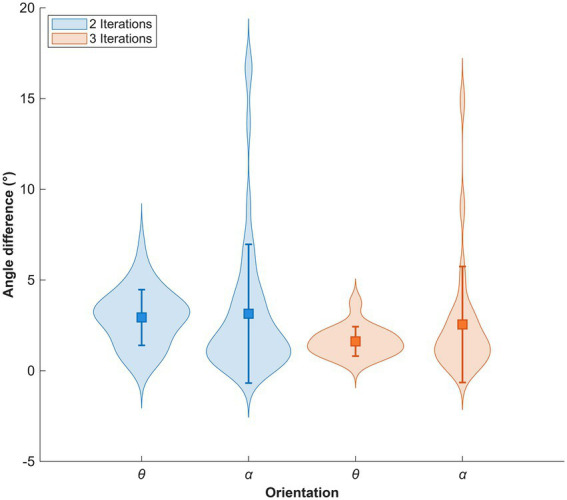
Validation of orientation resolution in anisotropic granulometry using 50 synthetic fibers. Violin plots show angular differences between prescribed and granulometrically estimated orientations for azimuth (θ) and elevation (α) using two-iteration (87 orientations) and three-iteration generation (337 orientations) discretizations. Squares indicate mean values, and error bars represent the standard error of the mean.

The angular deviation between prescribed and estimated orientations was computed for each structure. As shown in the violin plots (Figure 3), increasing the number of discrete structuring-element directions substantially enhances angular precision. Transitioning from two to three iterations shifts both the mean and mode of the error distributions toward zero for both azimuth and elevation.

Furthermore, the angular error in α is systematically lower than in θ . This disparity arises because α spans a narrower angular range compared to θ . For a fixed discretization level, this smaller range results in a higher effective sampling density, thereby yielding finer angular resolution and reduced deviation.

The results indicate that, while increasing the orientation resolution of anisotropic structuring elements significantly refines granulometric precision, it simultaneously increases the computational cost. Both algorithmic iterations prove highly viable, maintaining a stable average angular deviation between 1° and 3°. While the broader search space for θ results in occasional outliers and higher variance, α exhibits a narrower distribution. Notably, three-iteration discretization further tightens this error margin.

**Figure 4:**
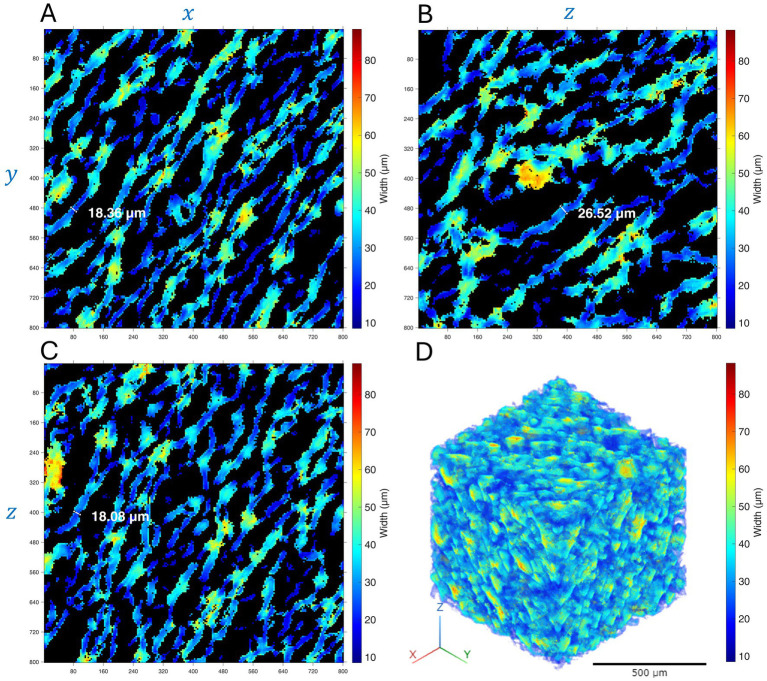
Granulometry-derived color maps illustrate the local fiber width distribution. The orthogonal cross-sections in the **(A)** *xy*, **(B)** *xz*, and **(C)** *yz* planes demonstrate high spatial consistency and include representative manual measurements (labeled #1) used for quantitative validation. **(D)** A 3D volumetric reconstruction confirming the continuity and robust width estimation of the full fiber network.

To validate the accuracy of the granulometry-derived width mapping, fiber dimensions were first qualitatively assessed across three orthogonal planes. As shown in Figure 4, the color-coded width maps demonstrate high spatial consistency with the underlying grayscale fiber architecture. The 3D volumetric reconstruction (Figure 4D) further confirms the continuity of the fiber network, indicating that the algorithm effectively captures the complex 3D geometry.

Beyond global trends, the qualitative interpretation of the mapping was further illustrated by examining the dimensions of a representative fiber strand across its three-dimensional projections. Manual measurements were performed on a representative fiber strand in each of the three orthogonal views. The individual manual measurements align closely with the range indicated by the local color-coding in the automated map. This strong correlation between the discrete manual points and the continuous automated mapping confirms the robustness of the granulometry-based approach for characterizing both individual fiber morphology and bulk material statistics.

The width estimates obtained from the three orthogonal views can be combined to derive the thickness of a planar fiber, enabling a direct geometric characterization of fiber cross-sectional dimensions.

**Figure 5:**
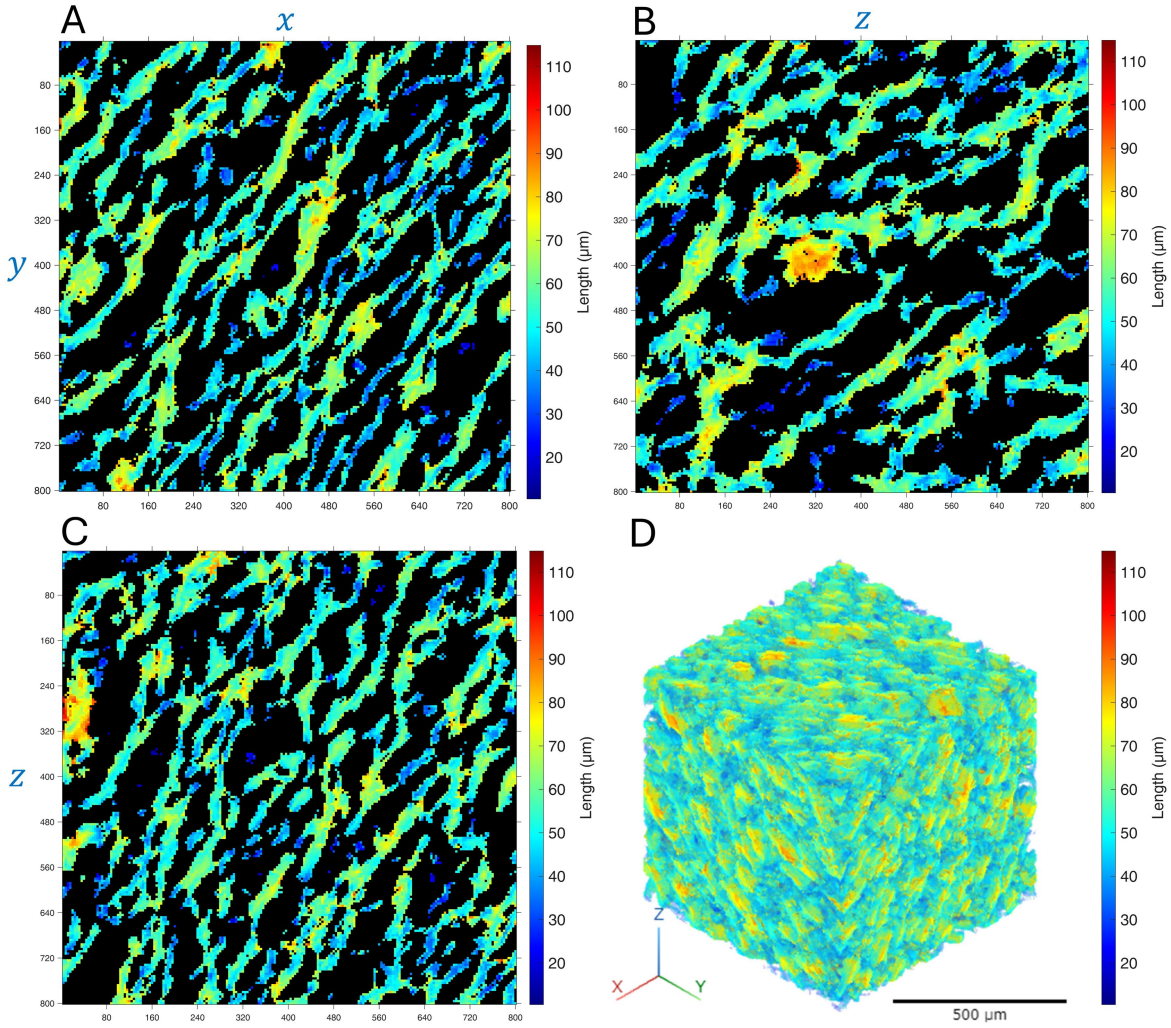
Granulometry-derived color maps illustrate the fiber length distribution. The orthogonal cross-sections in the **(A)** *xy*, **(B)** *xz*, and **(C)** *yz* planes demonstrate high spatial consistency. **(D)** A 3D volumetric reconstruction confirming the continuity and robust width estimation of the full fiber network.

The characteristic lengths were derived from an ellipsoidal approximation of the directional granulometric response distribution. The minor axis of the fitted ellipsoid provides a robust estimate of the transverse fiber thickness. In contrast, the major axis is more sensitive to deviations from an ideal ellipsoidal geometry and therefore does not directly represent the true physical fiber length. In particular, fiber bending, branching/interruption, voxel discretization, and partial truncation at the volume boundaries can bias the longitudinal estimate. Moreover, in regions where the microstructure exhibits a more planar or sheet-like organization and individual fibers are not clearly separable, the major-axis length reflects a dominant extension scale of the local fibrous assembly rather than the length of a single fiber. As shown in Figure 5, longer fibers are represented by warmer colors (toward red), whereas shorter fibers appear in cooler colors. Nevertheless, the major axis remains a meaningful descriptor of the preferred elongation and can be used to qualitatively compare relative fiber dimensions between samples or regions.

### 3.2 Sample analysis

#### 3.2.1 GMM clustering

**Figure 6:**
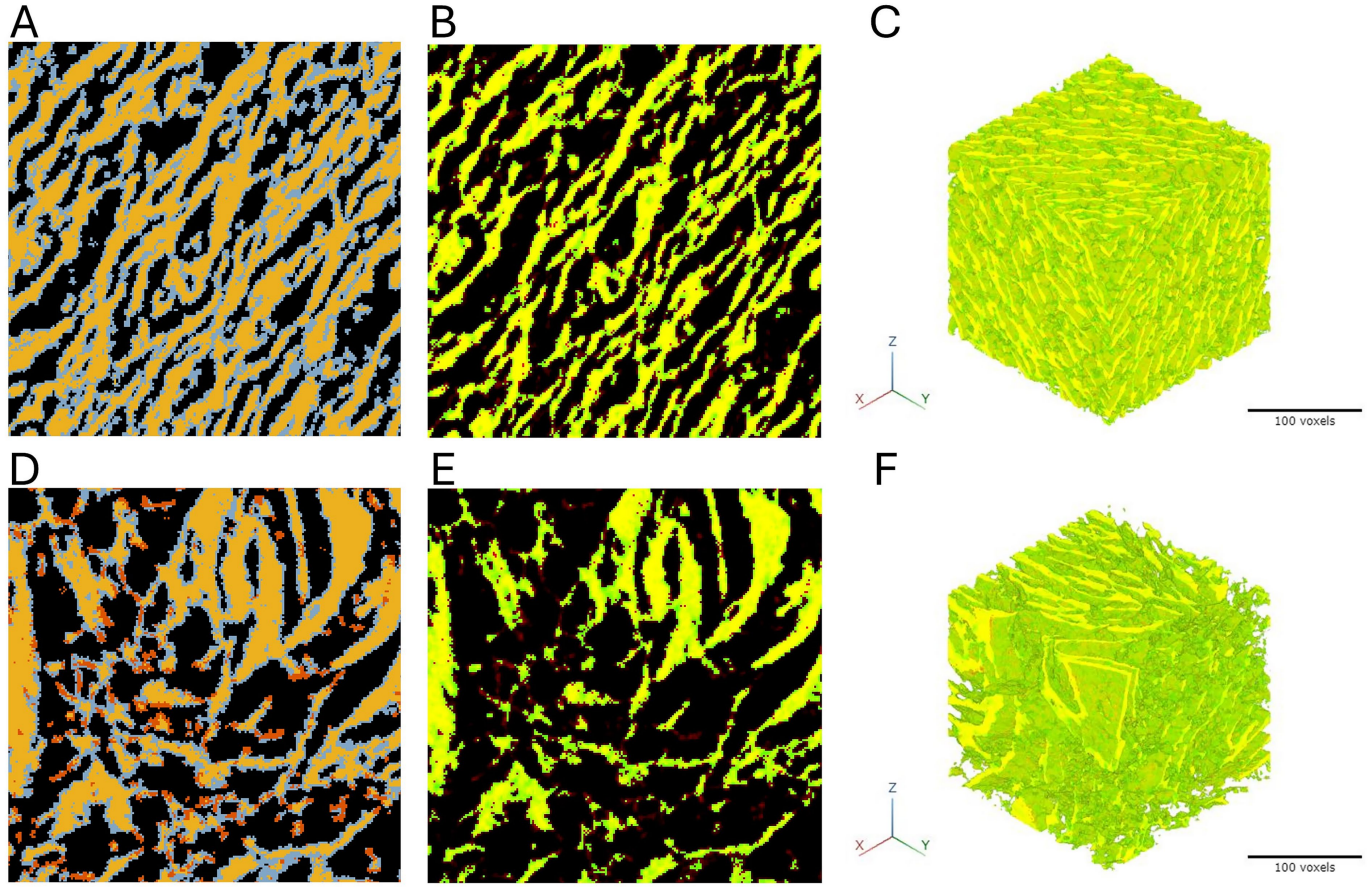
GMM clustering results for prototype sample 1 **(A–C)** and commercial sample 1 **(D–F)**, with overlap visualization. **(A,D)** Color-coded segmentation labels obtained by Gaussian Mixture Model (GMM) clustering. **(B,E)** Overlay of segmentation and original grayscale data: grayscale intensities are displayed using a red color gradient, the segmented target label (orange class) in green, and yellow indicates overlapping/co-localized regions. **(C,F)** 3D rendering of the overlay shown in **(B)** and **(E)**.

The prototype samples were composed solely of soy protein and water, whereas the commercial counterpart exhibited a more complex matrix containing soy protein, lipids, and flour-derived constituents, among others. To account for this, segmentation was performed using two classes for the prototype (Figure 6A) and three for the commercial sample (Figure 6D). The scattered red voxels in Figure 6D likely represent small lipid droplets within the commercial product. As illustrated in Figures 6B,E, and the 3D reconstructions in Figures 6C,F, the segmented voxels exhibit high spatial fidelity to the underlying grayscale features. Discrepancies are primarily confined to dark red voxels. Collectively, these results confirm that the proposed segmentation method robustly distinguishes foreground microstructures from the background phase.

The fiber morphology differed markedly between the prototype and commercial samples (Figures 6B,E). The prototype was characterized by elongated, relatively uniform fiber bundles with high aspect ratios and a pronounced directional alignment. In contrast, the commercial sample exhibited thicker and more irregular fibrillar structures. Although the commercial sample still showed an overall preferred orientation, its fiber distribution was less uniform and locally more random than that of the prototype. These observations indicate that the prototype exhibited greater anisotropy, whereas the commercial sample retained some structural orientation but with reduced directional consistency.

**Figure 7:**
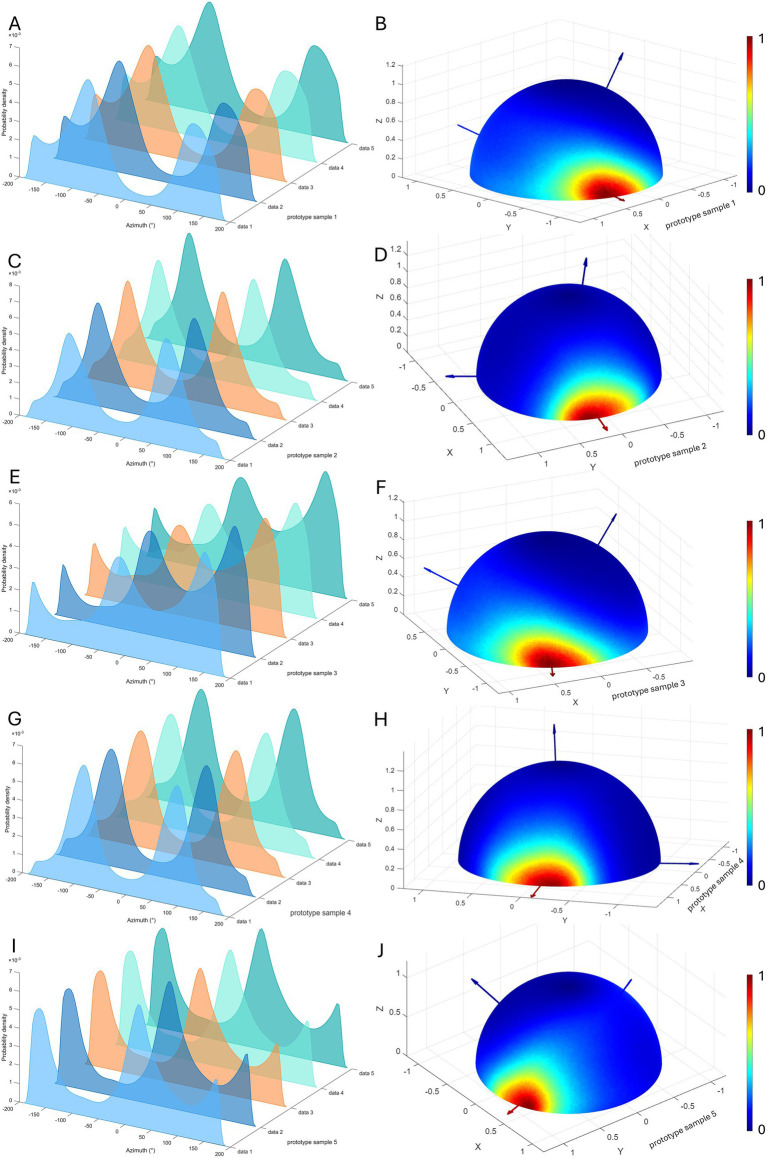
Fiber orientation distribution in prototype samples. For each prototype sample, KDE plots of azimuth angles from five repeated measurements are shown on the A,C,E,G,I, where each surface corresponds to one of five repeated measurements on the same sample. The corresponding 3D spherical projections of the orientation distribution are shown on the B,D,F,H,J, where each point on the upper hemisphere represents a unique fiber orientation defined by its elevation angle α (from the z-axis) and azimuth angle φ (in the xy-plane). Only the upper hemisphere is shown due to the head-tail symmetry of fiber orientations. Color on the sphere indicates the normalized density, ranging from blue (0) to red (1). The three arrows represent the eigenvectors of the orientation tensor, and their colors indicate the frequency of fiber orientations in the corresponding directions.

Despite these morphological differences, both samples exhibit a distinct laminar (layered) architecture, as evidenced by the 3D reconstructions in Figure 6C,F. However, the prototype shows a more uniform, well-defined layering, whereas the commercial sample shows layers that appear more irregular and fragmented. This higher degree of structural consistency in the prototype correlates with its uniform fiber distribution.

#### 3.2.2 Characteristics of fiber length and width

The fiber width distributions (Supplementary Figure S1) for all samples are consistently unimodal with a pronounced right-skewed profile. The long tail extending toward higher width values indicates the presence of a small proportion of considerably thicker fibers within the extrudate material.

Notably, prototype sample 1 (mode: 119.95 ± 1.01 μm) exhibited a fiber width (Table 2) comparable to that of sample 3, despite being processed at a lower extrusion temperature. This can be explained by the insufficient material temperature reached during extrusion, which likely did not exceed the critical threshold required for complete protein melting and subsequent texturization of the SPC. Högg et al. (34) demonstrated, using differential scanning calorimetry, that protein melting in SPC occurs at temperatures above 95 °C, and that for SPC hydrated to 60% moisture content, a material-temperature threshold of at least 120 °C is necessary to achieve significant protein denaturation and structure formation. This is consistent with the findings of Pietsch et al. (35), who used a closed cavity rheometer to analyze the rheological properties of SPC at 50% (w/w) moisture content and showed that the complex viscosity decreased exponentially with increasing temperature (50–120 °C). Below this threshold, the protein melt is characterized by high viscosity and limited molecular mobility, which restricts the extent of protein unfolding, cross-linking, and the development of well-defined anisotropic fiber structures (34).

**Table 2:** Characteristic fiber width of samples.

Sample name	Mode (μm)	Median (μm)	95% percentile interval (μm)
Prototype sample 1	119.95 ± 1.01	126.67 ± 0.99	[60.1, 217.1]
Prototype sample 2	112.35 ± 1.54	120.82 ± 1.16	[58.2, 211.8]
Prototype sample 3	119.45 ± 1.46	128.40 ± 0.40	[58.7, 228.8]
Prototype sample 4	124.95 ± 3.13	128.31 ± 1.01	[57.2, 228.4]
Prototype sample 5	138.92 ± 2.74	135.90 ± 1.69	[56.5, 243.5]
Commercial sample 1	182.23 ± 5.75	177.75 ± 3.20	[84.1, 279.5]
Commercial sample 2	140.37 ± 0.51	141.19 ± 2.28	[55.8, 241.6]
Commercial sample 3	154.69 ± 2.70	156.30 ± 3.26	[73.8, 243.7]

Data are presented as mean ± standard deviation of five repeated measurements.

Across the prototype samples, a progressive increase in fiber width (Table 2), was observed from sample 2 (mode: 112.35 ± 1.54 μm) to sample 5 (mode: 138.92 ± 2.74 μm), which corresponds to the stepwise increase in extrusion temperature applied during processing.

These observations are further corroborated by the work of de Boer et al. (36), who investigated the effect of high-moisture extrusion process conditions (100–160 °C, 50–70% moisture content) on the physical properties of SPC-based extrudates. They reported a significant positive correlation between processing temperature and projected particle area for SPC-based extrudates (*r* = 0.83, *p* < 0.05), with projected particle area increasing from 0.25 to 0.64 mm^2^ as the temperature was raised from 100 to 160 °C (at 60% moisture content). This progressive structural coarsening with increasing temperature is consistent with the fiber width increase observed in the present study and reflects the enhanced degree of protein denaturation and reorganization at higher processing temperatures.

Compared to the prototype samples, the commercial samples exhibited generally higher fiber width values. The broader distribution and larger fiber widths of the commercial samples could be attributed to differences in raw material composition (e.g., the commercial samples contained added wheat starch), pre-processing conditions, and extrusion parameters.

In contrast to the characteristic fiber width distributions, the characteristic fiber length distributions (Supplementary Figure S2) for all samples exhibit a unimodal and approximately symmetric profile. However, it should be noted that the characteristic fiber length provides only limited structural information. This limitation arises from three factors: first, the μCT image volumes were segmented into sub-cubes for analysis, meaning that fibers extending beyond the boundaries of a given sub-cube are truncated; second, the extrudate samples exhibit a predominantly planar, sheet-like structure, which may not accurately reflect the true length of individual fibers; and third, the measured length is also constrained by the structuring element length used in the granulometry analysis. Thus, relative comparisons among samples remain informative only to a certain degree.

Among the prototype samples, sample 1 (mode: 219.74 ± 1.23 μm) and sample 3 (mode: 229.57 ± 1.94 μm) exhibited comparatively higher characteristic fiber length (Table 3) values. For sample 1, this observation can be attributed to the insufficient extrusion temperature, which, as discussed in the preceding section, did not reach the critical threshold for complete protein melting. Under these conditions, the SPC material did not form well-defined individual fibrous structures but rather retained a continuous, planar sheet-like morphology. The absence of distinct fiber separation resulted in larger apparent fiber lengths, as the measurement captured continuous, unseparated protein strands rather than individual fibers.

**Table 3:** Characteristic fiber length of samples.

Sample name	Mode (μm)	Median (μm)	95% percentile interval (μm)
Prototype sample 1	219.74 ± 1.23	219.54 ± 1.11	[126.9, 312.7]
Prototype sample 2	215.86 ± 2.49	216.49 ± 1.60	[121.0, 314.2]
Prototype sample 3	229.57 ± 1.94	230.28 ± 1.06	[127.1, 334.2]
Prototype sample 4	215.85 ± 3.19	219.52 ± 1.37	[123.7, 323.9]
Prototype sample 5	212.98 ± 2.12	218.76 ± 1.06	[124.7, 329.6]
Commercial sample 1	272.92 ± 4.88	276.79 ± 3.72	[159.0, 404.3]
Commercial sample 2	240.67 ± 4.42	240.63 ± 4.54	[105.2, 369.4]
Commercial sample 3	249.87 ± 3.72	251.54 ± 2.98	[144.2, 362.6]

Data are presented as mean ± standard deviation of five repeated measurements.

For sample 3, the relatively higher characteristic fiber length may be attributed to the less well-defined and less uniform orientation of the fiber layers, as well as the relatively compact structure of the extrudate. The combination of a compact morphology with less directionally consistent layered structure may have resulted in larger apparent fiber lengths during the granulometry analysis.

Sample 5 (mode: 212.98 ± 2.12 μm), which was processed at 170 °C, exhibited the lowest characteristic fiber length among the prototype samples. This may be attributed to the degradation of protein molecular chains and the breaking of intermolecular bonds at temperatures exceeding 160 °C, which is detrimental to fibrous structure formation (37, 38).

The commercial samples exhibited generally higher and broader characteristic fiber length values (Table 3) compared to the prototype samples. As with the characteristic fiber width (Table 2), these differences could be attributed to variations in raw material composition (e.g., the inclusion of wheat starch in the commercial formulations), pre-processing conditions, and extrusion parameters.

Overall, the characteristic size obtained from the granulometry-based analysis can provide structural information about the fibers. In particular, the characteristic fiber width, which is less affected by sub-cube truncation and the planar sample geometry, can be interpreted as the thickness of the fiber layer.

#### 3.2.3 Orientation of fiber and anisotropy of the sample

**Figure 8:**
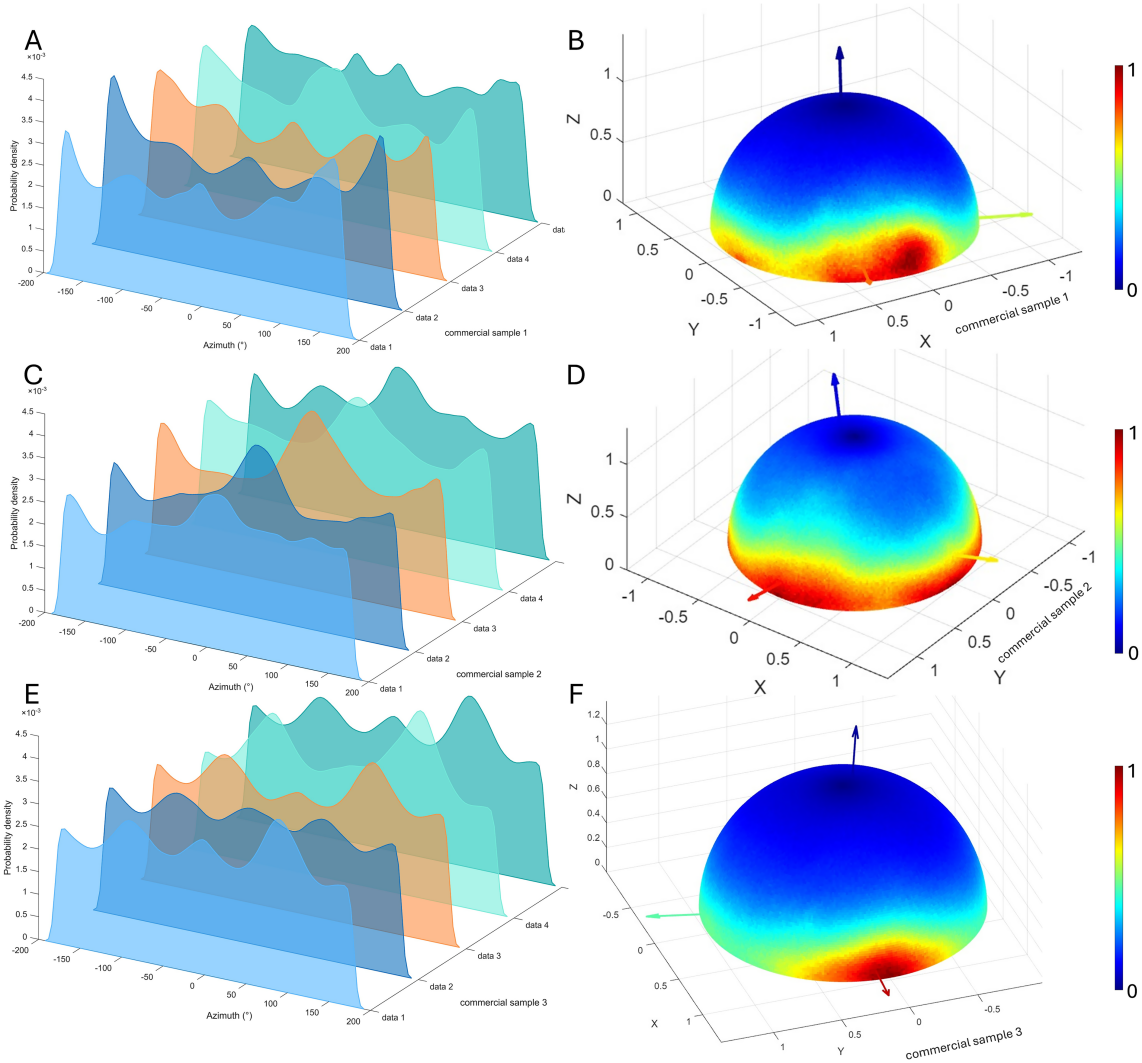
Fiber orientation distribution in commercial samples. For each commercial sample, KDE plots of azimuth angles from five repeated measurements are shown on the A,C,E, where each surface corresponds to one of five repeated measurements on the same sample. The corresponding 3D spherical projections of the orientation distribution are shown on the B,D,F, where each point on the upper hemisphere represents a unique fiber orientation defined by its elevation angle α (from the z-axis) and azimuth angle φ (in the xy-plane). Only the upper hemisphere is shown due to the head-tail symmetry of fiber orientations. Color on the sphere indicates the normalized density, ranging from blue (0) to red (1). The three arrows represent the eigenvectors of the orientation tensor, and their colors indicate the frequency of fiber orientations in the corresponding directions.

Figures 7, 8 present the orientation distributions of the fibers for the prototype and commercial samples, respectively. As evidenced by the 3D structural visualizations in Supplementary Figure S3, all samples exhibit a predominantly layered, sheet-like fiber architecture. Consequently, the elevation angle of the fibers provides limited discriminative information, as the fiber layers are oriented largely parallel to the sample plane. This is confirmed by the 3D spherical projections of the orientation distribution, where the elevation distribution is broad across the height of the hemisphere, whereas the azimuth distribution is comparatively narrow for the prototype samples. It should be noted that the azimuth angle ranges from −180° to 180°, while the elevation angle ranges from 0° to 90°.

The shape of the azimuth distribution reflects the orientation of the fiber layers. The eigenvector plane derived from the orientation tensor represents the dominant fiber layer direction. For example, Figure 7D shows an ellipsoidal distribution tilted toward a specific direction, indicating that the sheet-like fiber structure is inclined at a certain angle relative to the horizontal plane. This can be corroborated by the corresponding 3D structure of prototype sample shown in Supplementary Figure S3. The concentration of the azimuth distribution also provides information about the directional consistency of the fiber layers and, to a certain extent, reflects the degree of anisotropy within the extrudate.

It should be noted that the kernel density estimation (KDE) plots, which provide a nonparametric estimate of the underlying probability density function for the discrete angular data (Figures 7, 8, left panels), exhibit two peaks. This bimodality arises from the angular symmetry inherent to the orientation definition: fibers oriented at an azimuth angle α are physically equivalent to those oriented at α ± 180°, resulting in symmetric peak pairs in the KDE distribution.

Among the prototype samples, samples 4 and 5 exhibited relatively narrow azimuth distributions with correspondingly sharper KDE peaks, indicating a higher degree of directional uniformity of the fiber layers. This is consistent with the 3D structural observations in Supplementary Figure S3, where samples 4 and 5 display clearly defined, well-aligned layered fiber structures with uniform fiber orientation. In contrast, samples 1, 2, and 3 all exhibit layered structures, but with notable differences: sample 2 shows a relatively fragmented morphology; sample 1 displays a very compact, dense structure; and sample 3 exhibits more clearly defined fiber layers compared to samples 1 and 2, yet with less uniform fiber orientation than samples 4 and 5. Overall, the prototype samples show markedly more concentrated azimuth distributions than the commercial samples.

Among the commercial samples, sample 2 shows the broadest azimuth distribution with no clearly defined main direction, which corresponds to the nearly absent fiber layer structure observed in Supplementary Figure S3. In contrast, commercial sample 3 exhibits a relatively concentrated azimuth distribution, and the corresponding 3D structure in Supplementary Figure S3 confirms the presence of fiber layers with a relatively consistent orientation. Commercial sample 1 displays intermediate behavior. The generally broader azimuth distributions and lower directional consistency of the commercial samples compared to the prototype samples suggest less well-defined fibrous structures, which may be attributed to the more complex ingredient compositions (e.g., the inclusion of wheat starch) used in the commercial formulations.

**Figure 9:**
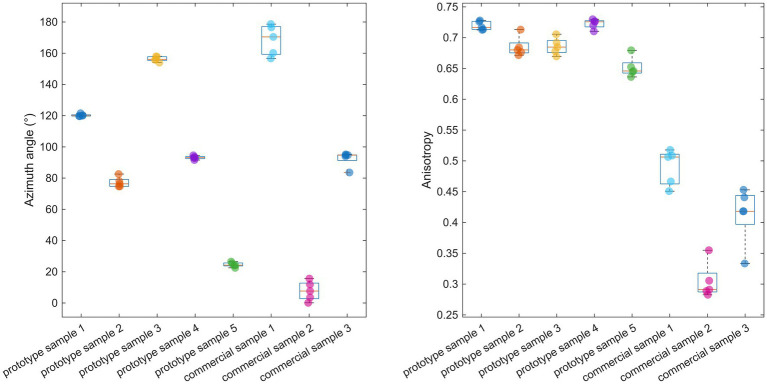
Main fiber orientation azimuth angle and anisotropy in prototype and commercial samples. Five repeated measurements were conducted for each sample and are shown as individual data points overlaid on the boxplots. **(A)** Main fiber-orientation azimuth angle (°). The repeated measurements of the prototype samples are tightly clustered, whereas some commercial samples show relatively greater variation between repetitions. **(B)** Anisotropy. The prototype samples exhibit relatively high and consistent anisotropy values, around 0.65–0.72, indicating pronounced directional alignment. In contrast, the commercial samples show lower and more variable anisotropy values, around 0.30–0.50.

Figure 9A presents the dominant azimuth angle for each sample, where the values correspond to the peak positions in the KDE distributions shown in Figures 8, 9. The prototype samples show notably less inter-replicate variability than the commercial samples, indicating a more uniform and stable fiber architecture. Among the prototype samples, samples 1, 4, and 5 exhibit particularly low inter-replicate variability, reflecting a more consistent structural formation. For the commercial samples, sample 3 shows the smallest variability. These observations are in agreement with the KDE distributions: narrower distributions correspond to smaller inter-replicate differences, which can also be recognized from the consistency of the KDE curve shapes across the different data replicates.

Figure 9B shows that the prototype samples exhibit consistently higher anisotropy values than the commercial samples. This is expected, given the more uniform, stable, and clearly defined fibrous structures observed in the prototype extrudates. Among the prototype samples, sample 4 shows the highest anisotropy, followed by samples 1 and 5, while sample 3 exhibits higher anisotropy than sample 2. This ranking is consistent with the structural observations in Supplementary Figure S3. sample 2 displays a relatively fragmented fiber morphology; sample 3 shows clearly defined fiber layers but with less uniform directionality; and sample 4 exhibits well-aligned, uniformly oriented fiber layers.

The relationship between extrusion temperature and anisotropy is consistent with previous findings in the literature. Grabowska et al. (39) reported that shearing SPC (45% dry matter) at 140 °C produced clearly defined layered structures containing coarse fibers, whereas processing at 120 °C yielded fragile structures with low mechanical strength, and processing at 130 °C produced harder structures with some layered features. Similarly, Högg et al. (34) reported a minimum texturization temperature of approximately 118 °C for SPC at 60% moisture content. Wittek et al. (40) also observed that increasing the material temperature from 124 °C to 135 °C led to enhanced anisotropy in extruded soy protein isolate (SPI). These findings collectively support the progressive increase in anisotropy observed from prototype sample 2 to sample 4 in the present study.

In contrast, prototype sample 5, which was processed at the highest temperature (170 °C), exhibited lower anisotropy than sample 4. This can be attributed to the excessively high processing temperature, which may cause the disruption of chemical bonds within the protein structure (41). Similarly, Chao et al. (42) observed dense fibrous filaments and meat-like structures in extrudates processed at 150 °C with 60% moisture content; however, when the temperature was increased to 180 °C, the extrudates formed rigid sheet-like structures with significantly reduced fibrousness. Pietsch et al. (35) also reported that higher temperatures do not necessarily result in greater anisotropy. These findings suggest that there is an optimal temperature window for achieving maximum fiber alignment and structural anisotropy during high-moisture extrusion of SPC.

Interestingly, prototype sample 1 also exhibited a relatively high anisotropy value and structural consistency, despite being processed at a temperature below the critical threshold for complete protein melting. As discussed in the preceding sections, sample 1 did not form well-defined individual fibers but rather retained a compact, dense structure. However, a certain degree of structural directionality is still observable in Supplementary Figure S3. This may be attributed to the formation of a multiphase system through phase separation, even at sub-optimal temperatures. Wittek et al. (43) demonstrated that a multiphase system, consisting of a water-rich dispersed phase surrounded by a protein-rich continuous phase, was already present in the screw section of the extruder, and that such a multiphase morphology could develop even at material temperatures as low as 95 °C. The directional alignment of these phase-separated domains under the shear flow in the cooling die may account for the relatively high apparent anisotropy observed in sample 1, even though true fiber formation had not yet occurred.

It should be noted that the absolute values of the reported orientation angles are relevant to how the sample is positioned within the CT scanner. In addition, rotation operations were applied during preprocessing to improve segmentation quality, further altering the absolute angle values. Therefore, the absolute orientation angles are not suitable for direct comparison across samples in this study. Instead, the relevant quantity is the dispersion of the orientation distribution, which characterizes fiber alignment independently of the initial sample placement.

#### 3.2.4 Shape characterization of fiber anisotropy

**Figure 10:**
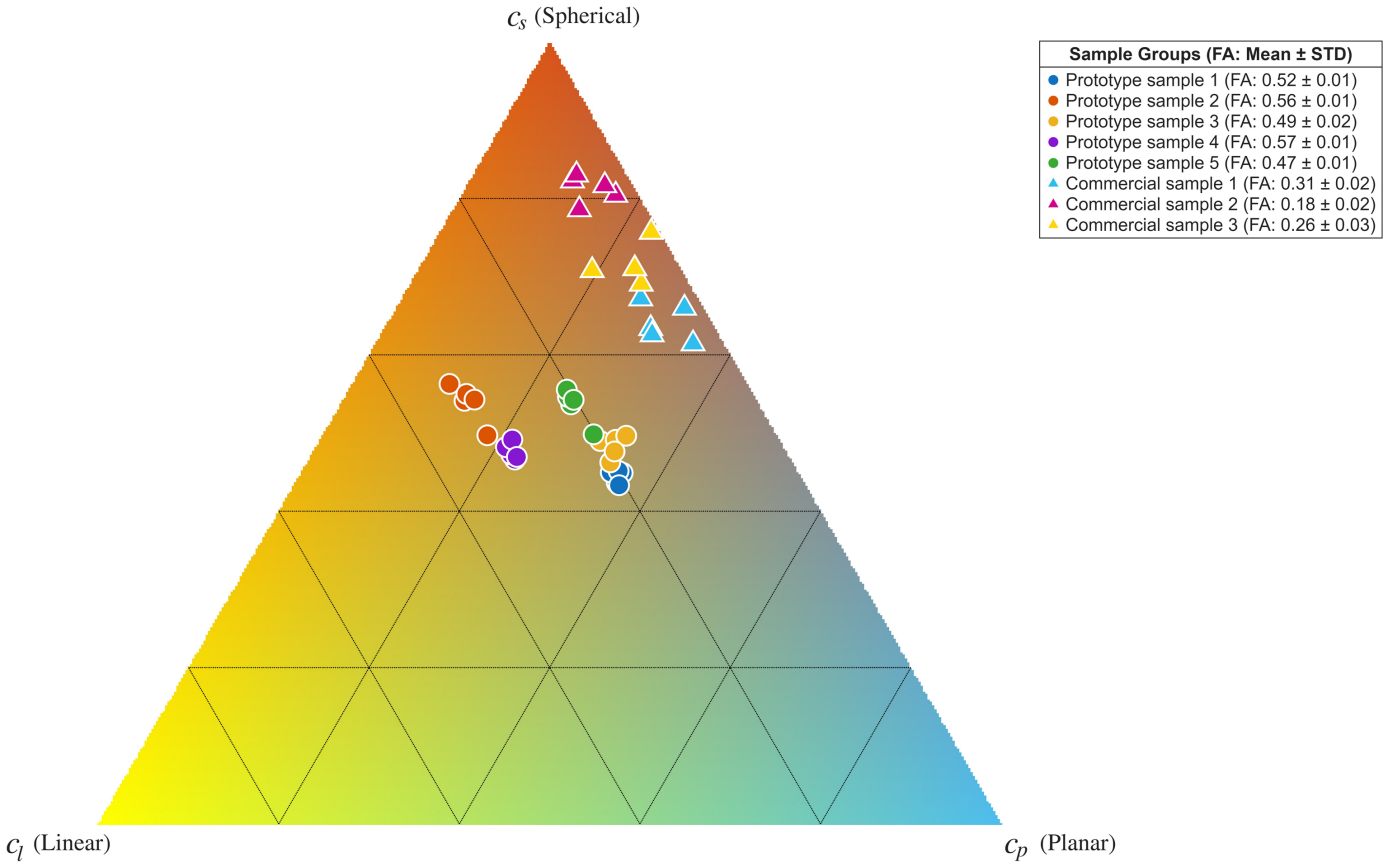
Ternary plots of FA. Each plot shows the distribution of samples in the anisotropy space defined by *c*_*l*_ (linear, yellow), *c*_*p*_ (planar, blue), and *c*_*s*_ (spherical, red), where each vertex represents the condition in which the corresponding component equals 1 (e.g., *c*_*l*_ = 1 for purely linear anisotropy). Five repetitions were conducted for each sample.

To further characterize the anisotropy and the shape of the fiber structures, FA and the normalized shape metrics $c_{l}$ (linear), $c_{p}$ (planar), and $c_{s}$ (spherical) were derived from the principal eigenvalues of the orientation tensor. In the Westin ternary diagram (Figure 10), the commercial samples are located in the upper region, indicating a higher spherical (isotropic) component.

Consistent with the preceding analyses, the commercial samples are located in the upper region of the Westin ternary diagram, indicating a higher spherical (isotropic) component. Commercial sample 2 is positioned closest to the cs vertex (FA: 0.18 ± 0.02), confirming its near-isotropic structure. In contrast, the prototype samples are positioned in the central region of the diagram, exhibiting relatively good anisotropy with a balance between linear and planar components.

Among the prototype samples, sample 4 exhibits a slightly higher linear component ( $c_{l}$ ) compared to the other samples, suggesting a more pronounced fiber-like structural character, which is consistent with the well-aligned fiber morphology observed in Supplementary Figure S3. Samples 1 and 3 show a higher planar component ( $c_{p}$ ), with sample 1 exhibiting the most pronounced planar character. This is in agreement with the compact, sheet-like morphology of sample 1 observed in Supplementary Figure S3, where the material retained a continuous planar structure rather than forming individually separated fibers.

However, it is important to note that the position within the Westin ternary diagram reflects the relative proportions of linear, planar, and spherical components, but does not directly indicate the absolute degree of anisotropy. For this, the FA value must be considered jointly. For instance, although sample 1 is positioned in the lower region of the diagram (high planar component), its FA value (0.52 ± 0.01) is lower than that of sample 4 (FA: 0.57 ± 0.01), which shows the highest FA among all samples. This indicates that while the structure of sample 1 is predominantly planar in character, the overall degree of anisotropy is less pronounced than in sample 4, where well-defined, directionally consistent fibers contribute to both a favorable shape and a high FA value.

Sample 5, which was processed at the 170°C extrusion temperature, is shifted toward the cs vertex compared to the other prototype samples. This can be attributed to the larger characteristic fiber width of sample 5, which leads to a more isotropic appearance in the shape analysis. Comparing sample 2 and sample 4, sample 4 exhibits a more pronounced planar structure, which is consistent with the clearer layered morphology of sample 4 observed in Supplementary Figure S3, whereas sample 2 displays a more fragmented microstructure.

## 4 Conclusion

In this study, a granulometry-based analysis was employed to characterize the 3D fiber structure of soy protein samples. The accuracy of the orientation estimation was validated using synthetically generated structures with known orientations, demonstrating that the method is robust with a mean angular error between 1° and 3°. The three-iteration yielded higher accuracy but at the cost of increased computational time. Additionally, the characteristic width and length were validated against the synthetic structures. The characteristic width showed good agreement with the defined dimensions, whereas the characteristic length provided only relative values and did not accurately reflect the true fiber length. Based on this validated framework, the characteristic fiber width, length, orientation, anisotropy, and shape were systematically investigated for both prototype and commercial extrudates.

The commercial samples, due to their more complex ingredient compositions (e.g., the inclusion of wheat starch), exhibited generally coarser and less well-defined fibrous structures with lower anisotropy and broader orientation distributions compared to prototype samples. In contrast, the prototype samples displayed relatively well-defined fibrous structures. With increasing extrusion temperature (from 120 to 170 °C), the characteristic fiber width increased progressively, while the anisotropy first increased and then decreased, indicating the existence of an optimal temperature window for achieving maximum fiber alignment and structural anisotropy during high-moisture extrusion of SPC. Notably, prototype sample 1, processed at 90 °C, did not form individually separated fibers but nevertheless exhibited a high degree of anisotropy and structural uniformity, which was attributed to the formation of a multiphase system through phase separation at sub-optimal temperatures.

The orientation distribution analysis proved to be a valuable tool for extracting structural information from the extrudates, including the directional consistency of fiber layers and the relative degree of anisotropy. Furthermore, the Westin ternary diagram provided additional insight into the shape characteristics of the fiber structures, enabling the distinction between linear, planar, and isotropic morphologies.

The present work extends the application of 3D orientation analysis based on μCT imaging to the characterization of plant-based meat analog microstructures. The quantitative descriptors derived from this approach, including characteristic fiber size, orientation distribution, fractional anisotropy, and shape metrics, provide a comprehensive framework for evaluating the fiber architecture of extruded products. These descriptors could support process optimization and structure-guided product development, and may serve as a basis for correlating 3D microstructural features with mechanical and textural properties in future studies.

## Data Availability

The raw data supporting the conclusions of this article will be made available by the authors, without undue reservation.
