# Supplementary material for: Three-dimensional characterization of fibrous microstructure in meat analogs using micro-computed X-ray tomography/μCT-based granulometry
Source: Front Nutr. 2026 Jul 22;13:1843070. doi: 10.3389/fnut.2026.1843070 (PMC13440075; doi:10.3389/fnut.2026.1843070)
Supplement: Supplementary file 1 [file Data_sheet_1.pdf]

Supplementary Material

1 Supplementary Figures and Tables

1.1 Supplementary Figures

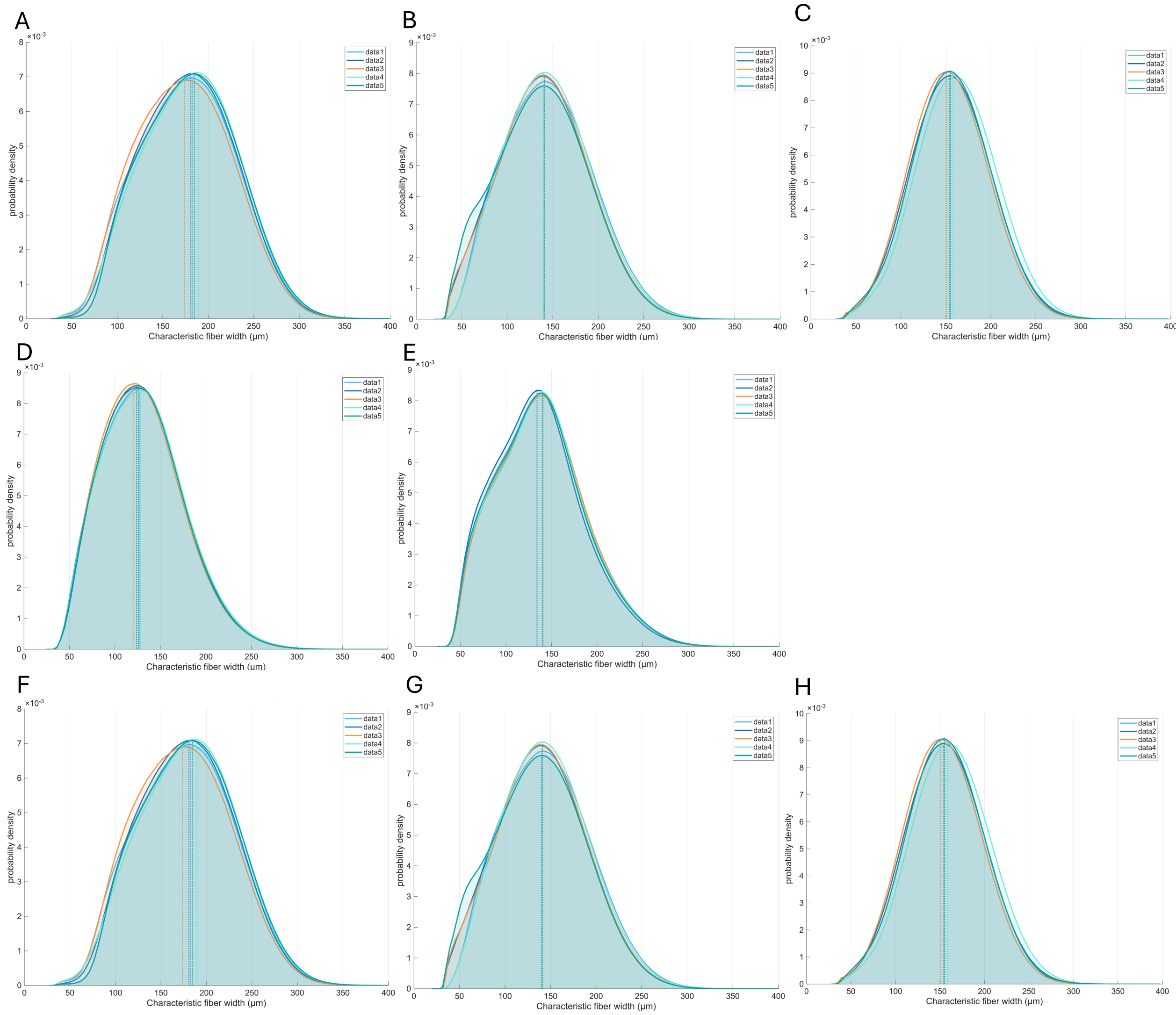

Figure S1. KDE of the characteristic fiber width distribution for prototype samples (A-E) and commercial samples (F-H). Dashed lines indicate the mode values. Each curve represents one sub-cube replicate (data 1-5).

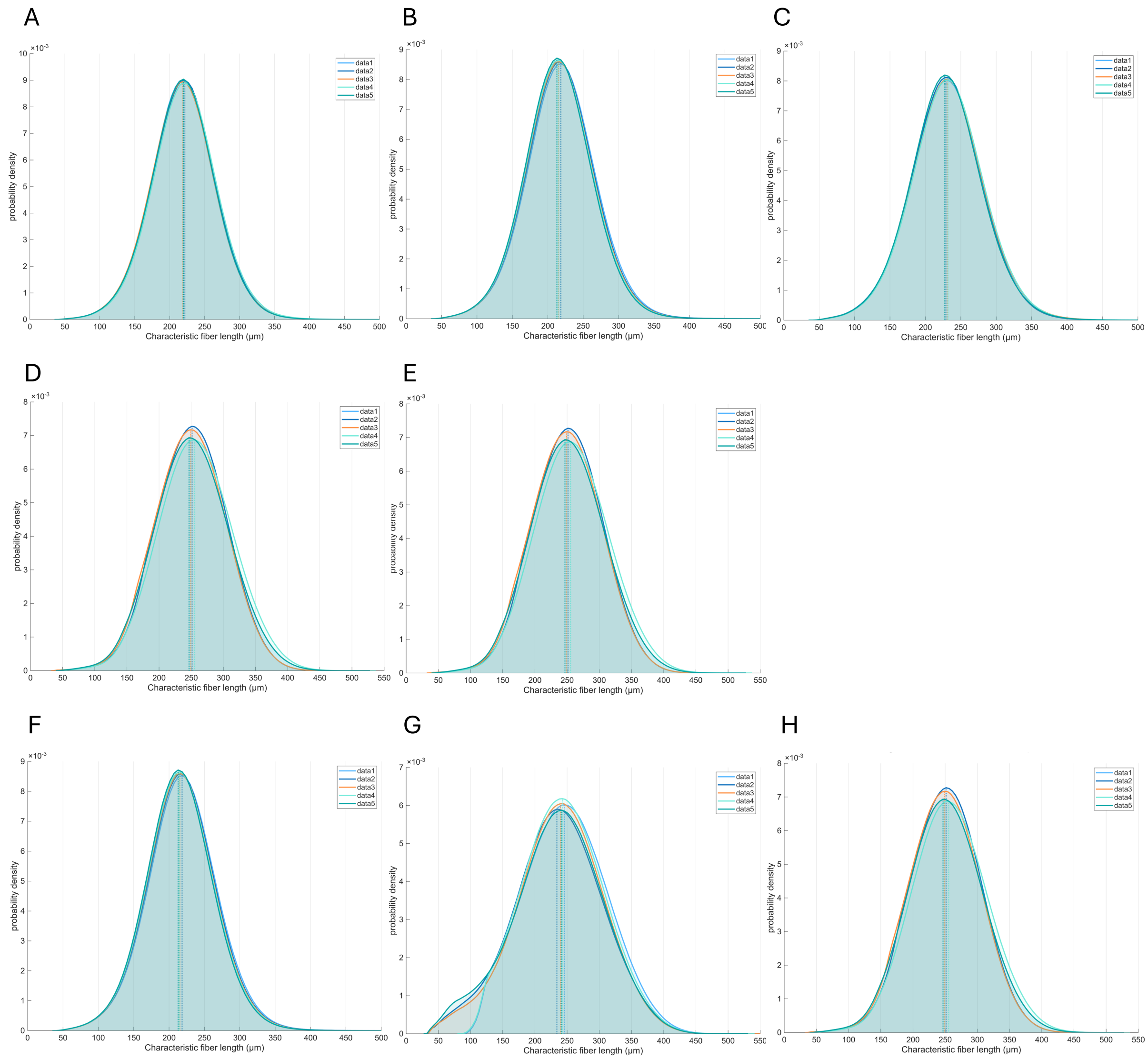

Figure S2. KDE of the characteristic fiber length distribution for prototype samples (A-E) and commercial samples (F-H). Dashed lines indicate the mode values. Each curve represents one sub-cube replicate (data 1-5).

Figure S3. Representative μCT cross-sectional images and 3D rendering of the segmented fiber structure. (A) xy-plane, (B) xz-plane, (C) yz-plane, and (D) 3D volume rendering. White regions represent the protein-rich fiber phase. Yellow crosshairs indicate the corresponding slice positions across planes.

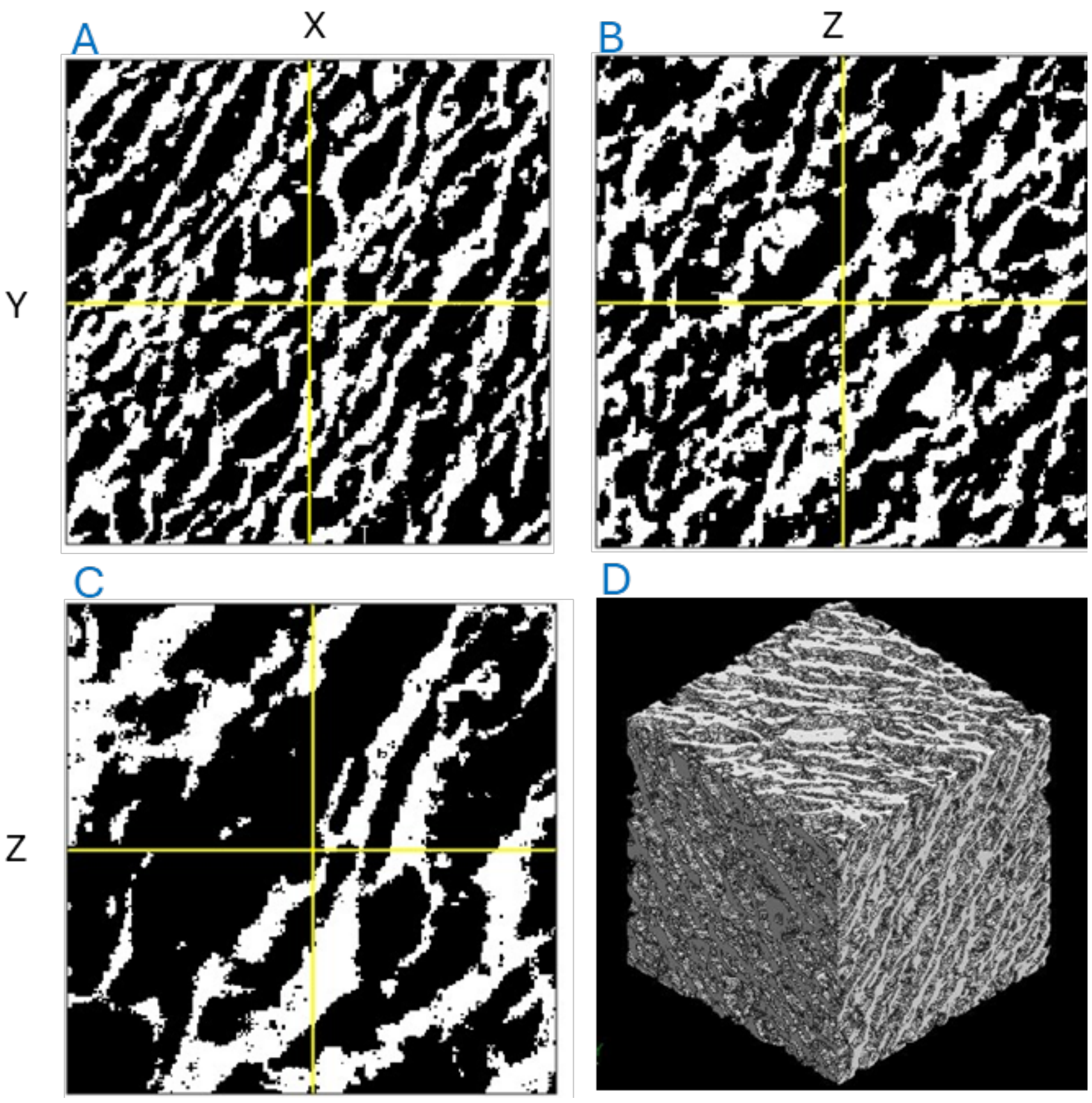

S3.1. Prototype sample 1

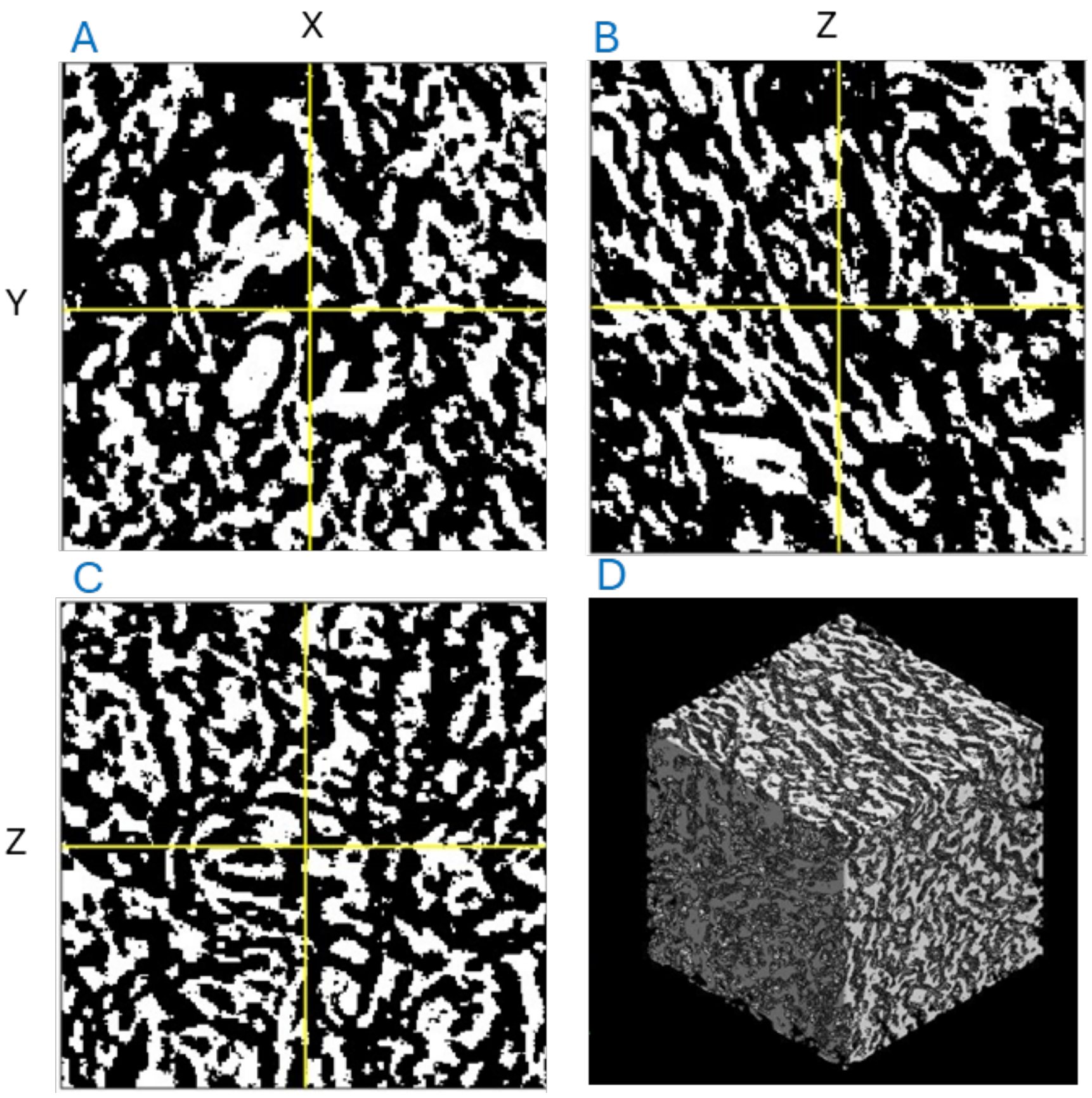

S3.2. Prototype sample 2

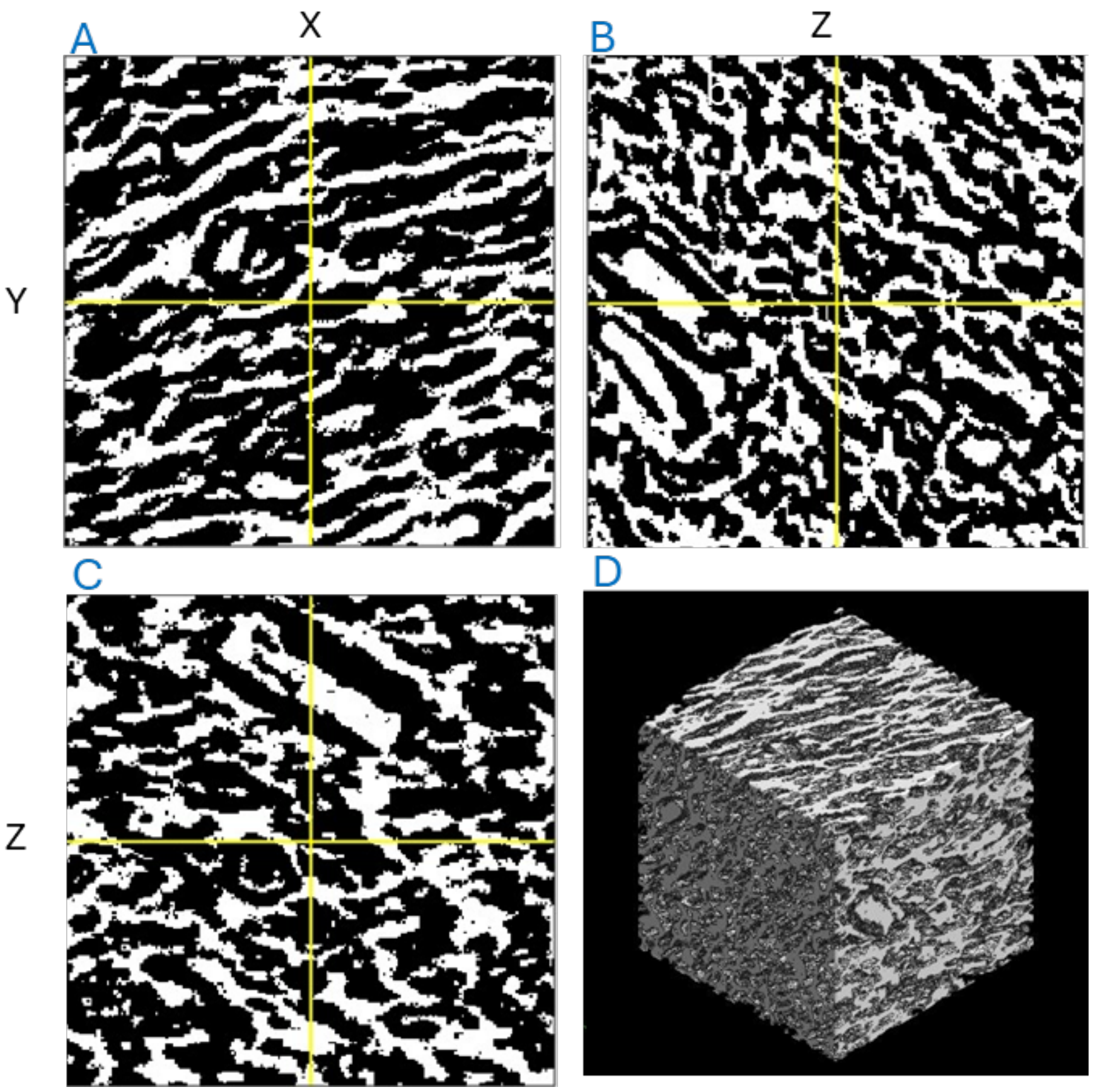

S3.3 Prototype sample 3

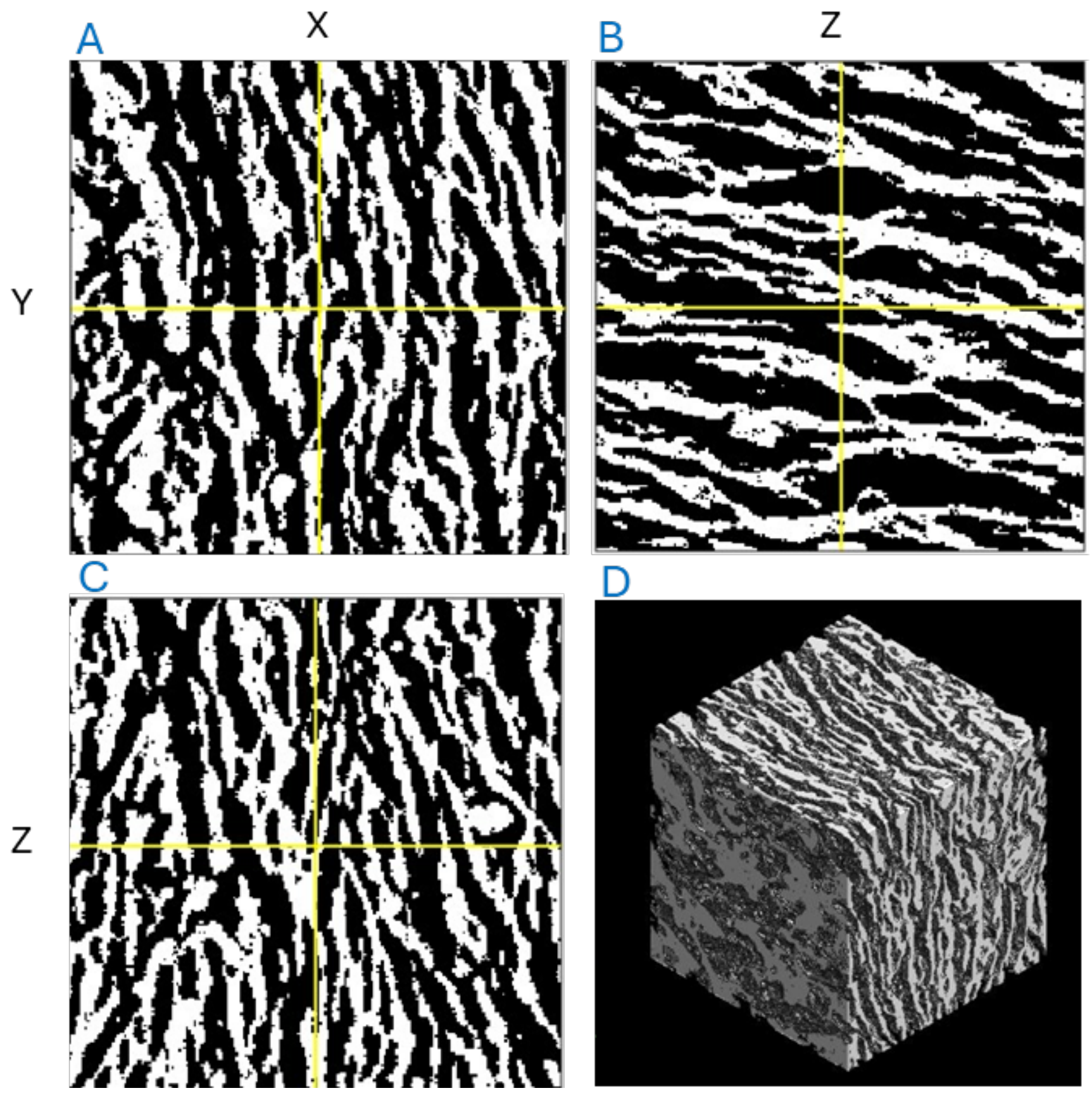

S3.4 Prototype sample 4

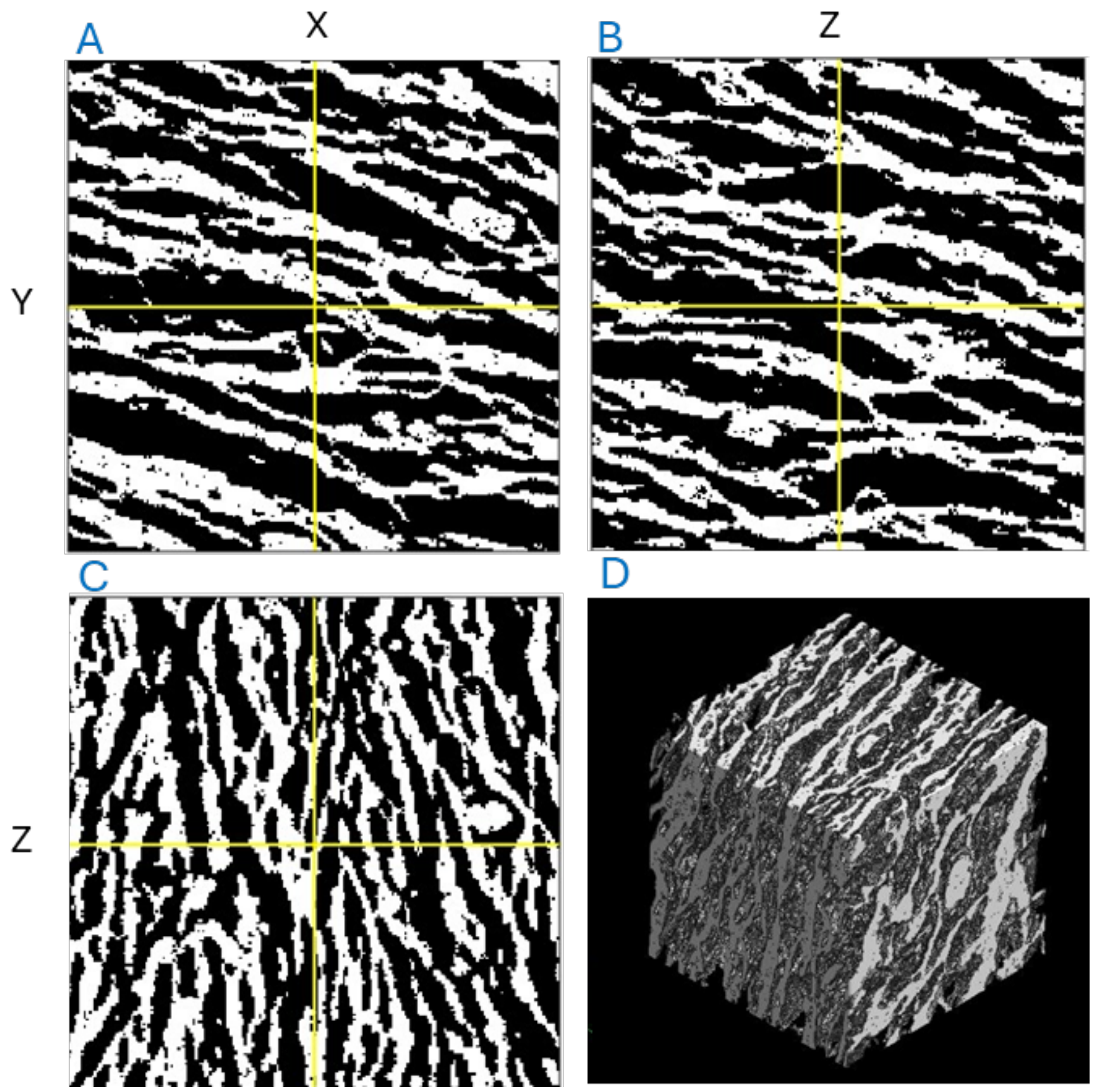

S3.5 Prototype sample 5

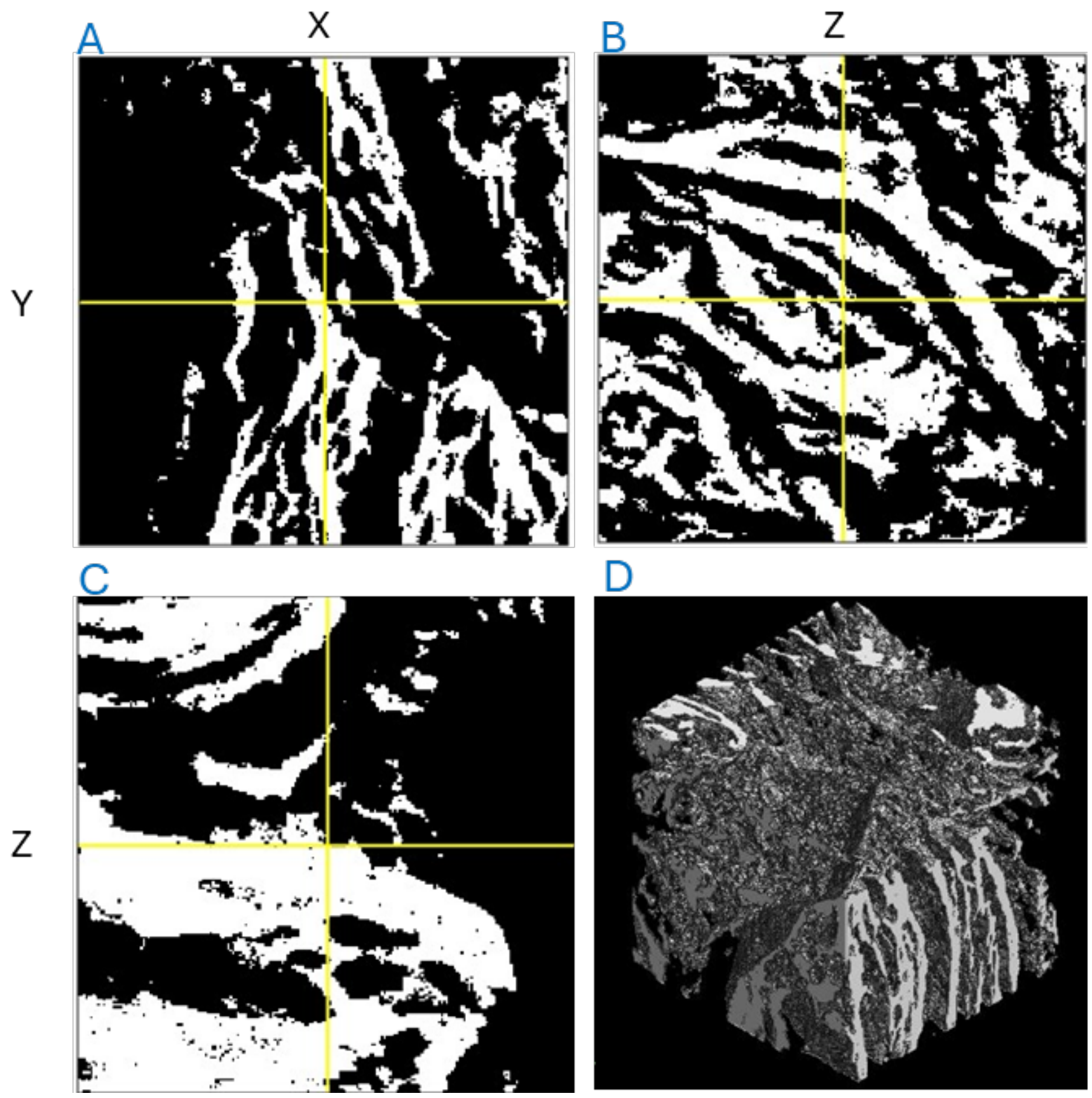

S3.6 Commercial sample 1

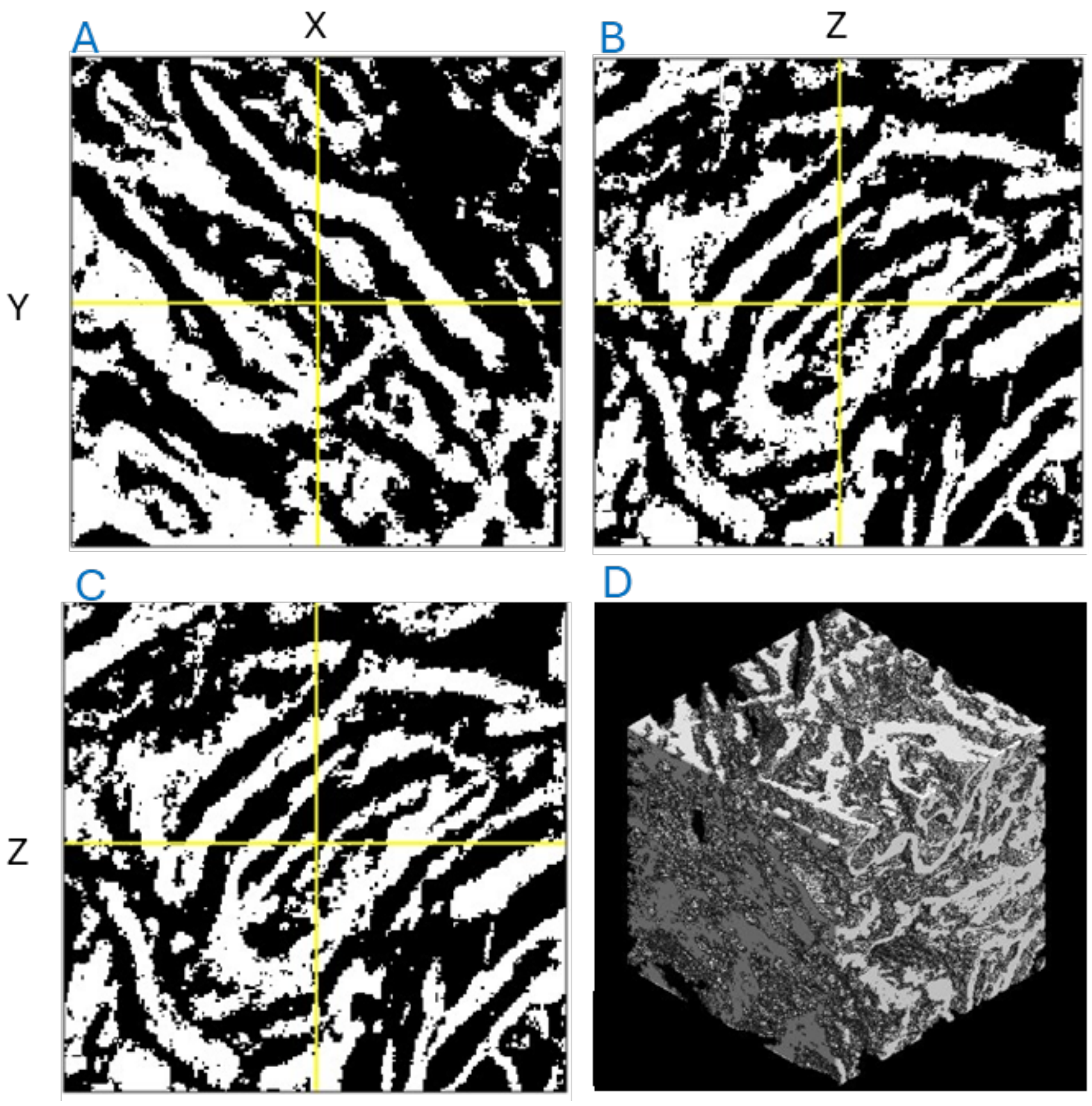

S3.7 Commercial sample 2

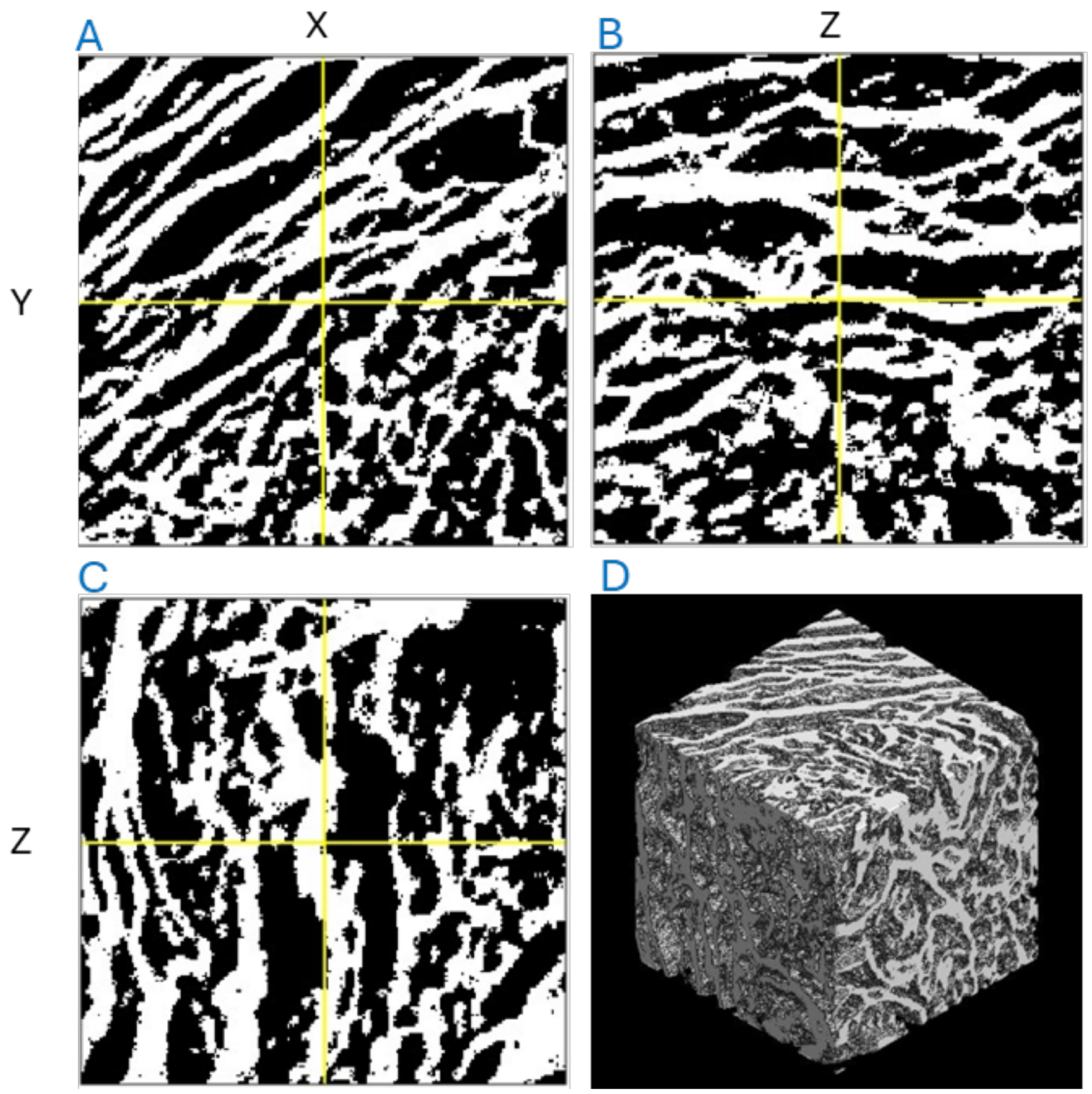

S3.8 Commercial sample 3

1.2 Supplementary Tables

	c_l	c_p	c_s	FA
prototype sample 1	0.2076	0.3559	0.4365	0.5233
	0.2076	0.3429	0.4495	0.5163
	0.1947	0.3562	0.4491	0.5089
	0.1978	0.3506	0.4515	0.5093
	0.2064	0.3603	0.4333	0.5244
prototype sample 2	0.3295	0.1077	0.5627	0.5550
	0.3236	0.1351	0.5413	0.5582
	0.3173	0.1328	0.5500	0.5497
	0.3118	0.1455	0.5427	0.5481
	0.3206	0.1824	0.4970	0.5734
prototype sample 3	0.1990	0.3113	0.4898	0.4889
	0.2017	0.3358	0.4625	0.5056
	0.1812	0.3276	0.4913	0.4768
	0.1671	0.3366	0.4964	0.4653
	0.1899	0.3338	0.4763	0.4907
prototype sample 4	0.3055	0.2286	0.4659	0.5749
	0.3062	0.2213	0.4724	0.5727
	0.3075	0.2106	0.4818	0.5697
	0.2956	0.2127	0.4917	0.5566
	0.3017	0.2288	0.4695	0.5706
prototype sample 5	0.2079	0.2556	0.5365	0.4695
	0.2021	0.2997	0.4982	0.4863
	0.2068	0.2471	0.5462	0.4634
	0.2033	0.2412	0.5555	0.4558
	0.2022	0.2553	0.5424	0.4622
commercial sample 1	0.0710	0.2952	0.6338	0.3227
	0.0632	0.2652	0.6716	0.2912
	0.0209	0.3193	0.6598	0.2880
	0.0736	0.3007	0.6258	0.3297
	0.0340	0.3518	0.6141	0.3259
commercial sample 2	0.0245	0.1711	0.8044	0.1717
	0.0636	0.1139	0.8225	0.1790
	0.0742	0.1399	0.7859	0.2130

	0.0551	0.1146	0.8303	0.1670
	0.0308	0.1529	0.8163	0.1641
	0.0507	0.2390	0.7103	0.2563
	0.0093	0.2340	0.7568	0.2080
commercial sample 3	0.0534	0.2561	0.6905	0.2728
	0.0989	0.1931	0.7080	0.2852
	0.0507	0.2390	0.7103	0.2563

Table S1. Supplementary data for Figure 10, providing the exact c_l, c_p, and $c_s$values used for the ternary plot.
